# Sterically Hindered Phosphonium Salts: Structure, Properties and Palladium Nanoparticle Stabilization

**DOI:** 10.3390/nano10122457

**Published:** 2020-12-09

**Authors:** Daria M. Arkhipova, Vadim V. Ermolaev, Vasily A. Miluykov, Aidar T. Gubaidullin, Daut R. Islamov, Olga N. Kataeva, Valentine P. Ananikov

**Affiliations:** 1 N.D. Zelinsky Institute of Organic Chemistry, Russian Academy of Sciences, Leninsky Prospect, 47, 119991 Moscow, Russia; val@ioc.ac.ru; 2 Arbuzov Institute of Organic and Physical Chemistry, FRC Kazan Scientific Center, Russian Academy of Sciences, Arbusov Street 8, 420088 Kazan, Russia; ermolaev@iopc.ru (V.V.E.); miluykov@iopc.ru (V.A.M.); aidar@iopc.ru (A.T.G.); daut1989@mail.ru (D.R.I.); olga-kataeva@yandex.ru (O.N.K.)

**Keywords:** PdNPs, TEM, catalysis, Suzuki cross-coupling reaction, sterically hindered phosphonium salts

## Abstract

A new family of sterically hindered alkyl(tri-*tert*-butyl) phosphonium salts (*n*-C_n_H_2n+1_ with *n* = 2, 4, 6, 8, 10, 12, 14, 16, 18, 20) was synthesized and evaluated as stabilizers for the formation of palladium nanoparticles (PdNPs), and the prepared PdNPs, stabilized by a series of phosphonium salts, were applied as catalysts of the Suzuki cross-coupling reaction. All investigated phosphonium salts were found to be excellent stabilizers of metal nanoparticles of small catalytically active size with a narrow size distribution. In addition, palladium nanoparticles exhibited exceptional stability: the presence of phosphonium salts prevented agglomeration and precipitation during the catalytic reaction.

## 1. Introduction

Since the beginning of the century, an explosive growth of publications devoted to special organic salts has arisen [1]. Ionic liquids (ILs) are organic salts with a melting point below 100 °C based on ammonium, imidazolium, phosphonium, and other cations combined with a variety of counter-ions [2]. They have received particular interest due to their attractive features such as high variability of structure, limited volatility and flammability, broad temperature range of the liquid state, thermal and chemical stability, wide electrochemical potential window and tunable solubility and miscibility [3,4,5] as well as highly variable biological activity [6]. One extraordinary advantage of organic salts is a structural directionality that is induced through ionic interactions and hydrogen bonding. This supramolecular network becomes an “entropic driver” for the formation of well-defined nanosized objects [7] and complex hierarchical systems [8,9].

From the whole variety of cations, we have chosen the phosphonium one, since the phosphorus atom allows the creation of an organic salt with high steric hindrance directly around the cation charge. Tunable bulkiness around the phosphorus atom is a key property in palladium-catalyzed coupling reactions [10].

The systematic study of a range of phosphonium salts with normal alkyl substituents connected with ionic liquids has become widespread in various fields [11]. Medicine is an important research direction for the application of the ionic liquid [12]. Ionic liquids possess antitumor [13,14] and antiviral activity [15] and could serve as organic antimicrobial additives to the polymeric material [16]. Organic salts have also shown their benefits in material science. Applications have been found for them as lubricants [17], in ionic liquid-based polymer electrolytes for Li-ion batteries [18] and supercapacitors [19] and in electrode functionalization [20]. 

Nanoparticle stabilization for the transition metal catalysis is a key field of application of this class of organic salts [21,22]. Multidisciplinary research at the junction of these booming areas has been shown by the extensive growth of publications devoted to the stabilization of nanoparticles in ionic liquids [23]. 

Concerning the application of solvent-stabilized nanoparticles, cross-coupling reactions have become one of the most powerful and versatile tools in modern organic chemistry. It is not surprising that metal nanoparticles exhibiting activity in these reactions found such a response [24,25]. A large variety of nanoparticle preparation methods, different materials that can serve as supports or grafting cores, and several media have been studied and discussed [26]. Several studies have supported palladium nanocatalyst for cross-coupling reactions [27,28]. For example, in recent years, different types of nanoparticle support materials were investigated; for example, metal oxides [29,30,31], zeolites [32,33,34], carbons [35,36,37], ordered porous materials [38,39] and synthesized polymers [40] (e.g., ionic polymers [41]) and modified natural polymers [42,43]. There are several studies on mixed composition for palladium nanoparticle (PdNP) support [44,45,46,47], and the need for a rational design of catalyst support has been emphasized [48,49]. In pursuit of a rational approach to the creation of a catalytic system based on PdNPs, we have chosen phosphonium salts with a sterically hindered cation with a variable fourth alkyl chain.

Tri-*tert*-butylphosphine was first synthesized and described in 1967 by Hofmann and Schellenbeck [50]. The study of its chemical properties led to the synthesis the first member of sterically hindered phosphonium salts, tri-*tert*-butyl(methyl)phosphonium iodide.

A decade later, Schmidbauer et al. used tri-*tert*-butyl(methyl)phosphonium bromide as a precursor for tri-*tert*-butyl(methylene)phosphorene [51]. Originally, phosphonium salt was synthesized from tri-*tert*-butylphosphine and condensed bromomethane and characterized by physical–chemical methods. In their next study, these authors described the synthesis of the extreme sterically hindered tetra-*tert*-butylphosphonium tatrafluoroborate through a series of consequent phosphonium–phosphorane transformations [52]. Intermediate substances have been characterized with standard methods, and the structure of the desired product was confirmed with X-ray analysis. Two new salts—tri-*tert*-butyl(ethyl)phosphonium and tri-*tert*-butyl(*iso*-propyl) phosphonium bromides—have been described.

Srivastava and co-workers reported the synthesis of tri-*tert*-butyl(methyl)phosphonium iodide in the coordination sphere of Pt from (*t*-Bu_3_P)_2_Pt and iodomethane [53]. The reaction of [CpFe(CO)_2_CH_2_Br] with tri-*tert*-butylphosphine under mild conditions and following bromide–anion exchange reaction with BAr_4_^−^ were reported in 1989 [54].

Increasing interest in the field of sterically hindered phosphonium salts has continued in the last decade. A new method of sterically hindered salt synthesis by methylation of tri-*tert*-butylphosphane hydrobromide with dimethyl acetal has been suggested [55]. We have also described the synthesis of the sterically hindered ionic liquids tri-*tert*-butyl- and tricyclohexyl(decyl)phosphonium tetrafluroborates [56]. Furthermore, hindered phosphonium salts were conjugated with frustrated Lewis pairs ([ethylP^t^Bu_3_]^+^ [(C_6_F_5_)_3_B]_2_) [57].

This trend led to the emergence of systematic studies devoted to the variety of phosphonium salts [58,59]. In our lab, the first members of sterically hindered phosphonium salts have been synthesized and described [60,61]. 

Here, for the first time, we describe a systematic study of physical–chemical properties of phosphonium salts combined with the investigation of their influence on PdNP stabilization and the catalytic activity in practically demanding cross-coupling reaction.

## 2. Materials and Methods

### 2.1. Instrumental

**NMR spectroscopy.** NMR spectra were recorded on a Bruker MSL-400 instrument (BRUKER BioSpin GMBH am Silberstreifen, D-76287, Rheinstetten, Germany) at 20 °C (^1^H 400 MHz, ^31^C 100.6 MHz, ^31^P 161.7 MHz). SiMe_4_ was used as internal reference for ^1^H and ^13^C NMR spectra, and 85% H_3_PO_4_ as external reference for ^31^P NMR spectra.

**X-ray analysis.** Data sets for single crystals were collected on a Bruker AXS Kappa APEX Duo diffractometer (Bruker AXS GmbH, Karlsruhe, Germany). Programs used: data collection APEX2, data reduction SAINT, absorption correction SADABS version 2.10, structure solution SHELXT [62], structure refinement by full-matrix least-squares against F^2^ using SHELXL [63]. The hydrogen atoms were inserted at the calculated positions and refined as riding atoms. The positions of the hydrogen atoms of methyl groups were found using rotating group refinement with idealized tetrahedral angles. The figures were generated using Mercury 4.1 [64] program. 

**Electrospray Ionization Mass Spectrometry (ESI-MS).** The ESI-MS measurements were performed using an AmazonX ion trap mass spectrometer (Bruker Daltonic GmbH, Bremen, Germany) in positive (and/or negative) mode in the mass range of 70–3000. The capillary voltage was −3500 V, nitrogen drying gas—10 L·min^−1^, desolvation temperature −250 °C. A methanol/water solution (70:30) was used as a mobile phase at a flow rate of 0.2 mL/min by binary pump (Agilent 1260 chromatograph, Santa Clara, CA, USA). The sample was dissolved in methanol to a concentration of 10^−6^ g·L^−1^. The instrument was calibrated with a tuning mixture (Agilent G2431A, Santa Clara, CA, USA). For instrument control and data acquiring the TrapControl 7.0 software (Bruker Daltonik GmbH, Bremen, Germany) was used. Data processing was performed by DataAnalysis 4.0 SP4 software (Bruker Daltonik GmbH, Bremen, Germany).

**Thermogravimetric analysis (TGA) and Differential Scanning Calorimetry (DSC).** STA449 F3 (NETZSCH, Selb, Germany) were used for the thermal analysis (thermogravimetry/differential scanning calorimetry) in which the variation of the sample mass as a function of temperature and the corresponding heats are recorded. An approximately 7mg sample was placed in an Al (or Al_2_O_3_ at temperatures above 600 °C) crucible with a pre-hole on the lid and heated from 25 to 600 °C. The same empty crucible was used as the reference sample. High-purity argon was used with a gas flow rate of 50 mL/min. TG/DSC measurement were performed at the heating rates of 1–50 K/min.

**Transmission Electron Microscopy (TEM).** TEM images of the PdNPs before and after the reaction were obtained using a HT7700 (Hitachi, Hitachinaka-shi, Japan) transmission electron microscope. Accelerating voltage 40–120 kV; maximum magnification 800,000; resolution 0.144 nm.

**X-ray powder diffraction (XRPD).** XRPD measurements were performed on an automatic D8 Advance diffractometer (Bruker, Karlsruhe, Germany) equipped with a Vario attachment and Vantec linear PSD using Cu radiation (40 kV, 40 mA) monochromated by a curved Johansson monochromator (λ CuKα1 1.5406 Å). Room temperature data were collected in the reflection mode with a flat-plate sample. 

**Small angle X-ray scattering (SAXS).** SAXS data for samples were collected with the AXS Nanostar SAXS system (Bruker, Karlsruhe, Germany) using CuKα (λ 1.5418 Å) radiation from a 2.2 kW X-ray tube (40 kV, 35 mA) coupled with Gobbel mirrors optics and a HiStar 2D area detector. The beam was collimated using three pinholes with apertures of 1000, 400 and 750 μm. The instrument was operated with a sample-to-detector distance of 63.5 cm to provide data at angles 0.1° < 2θ < 4.8°, which correspond to 0.007 Å^−1^ < s < 0.34 Å^−1^. The value of s is proportional to the inverse of the length scale (s = (4π/λ) sin (θ) in units of Å^−1^). 

**Gas Chromatography–Mass Spectrometry (GC-MS).** The conversion of 1,3,5-triphenylbenzene was estimated by gas chromatography coupled with mass spectrometry on a gas chromatograph 5977A (Agilent, Santa Clara, CA, USA) using electron ionization with an energy of ionizing electrons of 70 eV, the temperature of the ion source 250 °C, and an ID-BP5X capillary column (analog of DB-5MS, length 50 m, diameter 0.32 mm, thickness of the phase layer 0.25 μm), and helium as a carrier gas. The mass spectral data were processed using the Xcalibur program. Prior to injection, a sample of the studied substance was dissolved in chromatographically pure ethanol in a concentration of ~10^−3^ g·μL^−1^ (volume of the injected sample 0.1 μL). The chromatographic regime was as follows: temperature of the injector 250 °C, flow split 1:10; temperature-programmed column: initial temperature 120 °C (1 min), then heating with a rate of 20 °C·min^−1^ to 280 °C, and final temperature 280 °C (15 min); the flow rate of the carrier gas through the column was 2 mL·min^−1^. The temperature of the communication device with the mass spectrometer was 280 °C. 

### 2.2. Materials

All procedures associated with the preparation of the starting reagents, synthesis, and isolation of products were carried out under inert atmosphere using the standard Schlenk technique. Tri-*tert*-butylphosphine (Dal-Chem, Nizhny Novgorod, Russia), ethyl iodide and *n*-alkyl bromide (Sigma-Aldrich, St. Louis, MO, USA), phenylboronic acid (Alfa Aesar, Heysham, Lancashire, UK), 1,3,5-tribromobenzene (Alfa Aesar), Pd(OAc)_2_ (Alfa Aesar), KOH (Himreaktiv, Nizhny Novgorod, Russia), NaBF_4_ (Himreaktiv, Nizhny Novgorod, Russia) analytical grade, without additional purification were used in the work. Detailed synthesis and characterization of the phosphonium salts described in Supplementary Materials.

#### 2.2.1. Typical Procedure for PdNPs Preparation

0.0004 g (0.00178 mmol) of palladium acetate and 0.178 mmol of phosphonium salt was dissolved in 9 mL ethanol and stirred during 20 min at room temperature. The color of solution changes from transparent to light brownish gray. 

#### 2.2.2. Typical Procedure for Suzuki Cross-Coupling

To the fresh solution of colloidal Pd 0.157 g (0.50 mmol) of 1,3,5-tribrombenzene, 0.276 g (2.25 mmol) of phenyl boronic acid, 0.128 g (2.25 mmol) of potassium hydroxide were added. Reaction mixture was stirred over 7 h at 30 °C. Organic compounds were extracted with 9 mL toluene and analyzed by GC-MS.

## 3. Results and Discussion

### 3.1. Synthetic Procedures 

**Scheme 1:**
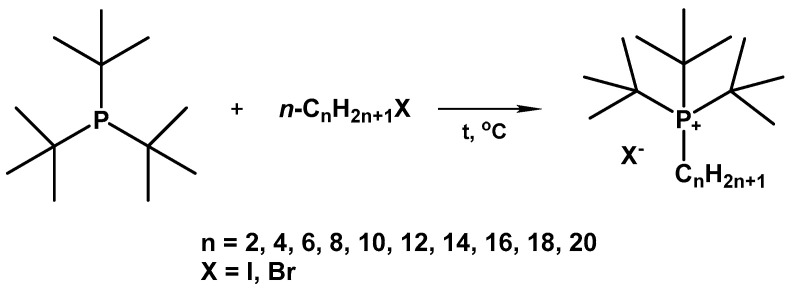
The quaternization reaction of tri-*tert*-butylphosphine and 1-haloalkane.

Phosphonium salts with halogen anions were obtained by nucleophilic addition of tri-*tert*-butylphosphine to respective linear 1-haloalkane (Hal = I or Br) according to the described procedure (Scheme 1) [65]. The quaternization reaction was conducted under solvent-free conditions except for the cases with solid haloalkanes (C_16_H_33_Br, C_18_H_37_Br, C_20_H_41_Br). For the solid reactants, we applied acetonitrile as a solvent due to its polar aprotic nature. The temperature of the reaction varied, depending on the applied haloalkane, from 0 °C for EtI to 90–110 °C for liquid 1-bromoalkanes and 80 °C for the solid ones. The reaction was ended after the disappearance of the phosphine signal in the ^31^P NMR spectrum. 

Compared to tri-*n*-butylphosphonium salts, the temperature for the synthesis of sterically hindered products was higher [58,59] even though the pK_a_ of Bu^n^_3_P (7.6) [66] is lower than that of Bu^t^_3_P (11.4) [67]. This fact could be explained by steric reasons.

**Scheme 2:**
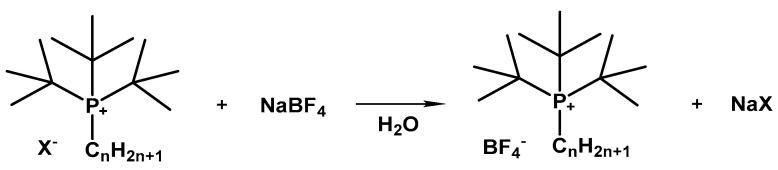
The anion exchange reaction.

Then phosphonium halides were put into a metathesis reaction with weakly coordinating anions, namely tetrafluoroborate (Scheme 2). The anion exchange reaction was carried out in water at room temperature. The resulting phosphonium salt was precipitated as soon as sodium tetrafluoroborate was added to the water solution of the phosphonium halide.

New compounds were obtained in high yields (up to 98%; see ESI for details). The phosphonium salts are white or yellowish crystalline or amorphous solids that are soluble in chloroform, dichloromethane, acetone, ethanol, methanol and almost insoluble in diethyl ether and petroleum ether.

### 3.2. NMR Spectroscopy 

#### 3.2.1. ^31^P NMR Spectra

The ^31^P{^1^H} NMR spectra of the alkyl(tri-*tert*-butyl) phosphonium halides (Table 1) each exhibited a singlet at 49.71 ± 0.41 ppm, with one exception, **1a**, where the signal was at 50.53 ppm. The ^31^P{^1^H} NMR spectra of the alkyl(tri-*tert*-butyl) phosphonium tetrafluoroborates all exhibited a singlet at 49.10 ± 0.07 ppm, with one exception, **1b**, where the signal was at 49.96 ppm. The chemical shifts of **1a** and **1b** appear in a lower magnetic field, possibly due to the lack of electron density of ethyl substituent to compensate for the cationic charge on the phosphorus atom.

Although tri-*tert*-butylphosphine is extremely sensitive to oxidation in air and flammability, it requires exceptional care in handling and an inert atmosphere to achieve high purity ^31^P{^1^H} NMR spectra of the phosphonium salts containing a single peak. 

#### 3.2.2. ^1^H NMR Signals for C(α)-H Protons

**Figure 1:**
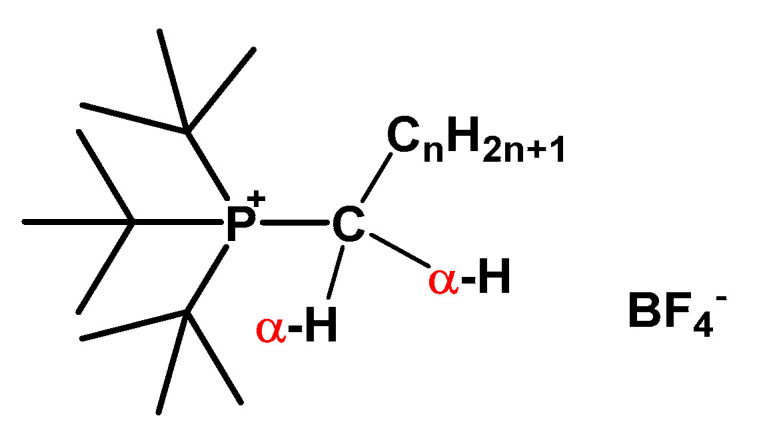
α-protons in the sterically hindered phosphonium salts.

We found that the α-protons are very sensitive to anion exchange (Figure 1). The chemical shift of α-protons of phosphonium tetrafluoroborates appears in higher magnetic fields (2.30 ± 0.03 ppm) than phosphonium bromides (2.61 ± 0.07 ppm) (Table 1). This is caused by the presence of hydrogen bonds between α-protons and bromide anion bearing high electron affinity, while the weakly coordinated tetrafluoroborate anion interacts with the surrounding ions via non-covalent Coulomb forces [56].

The exception is α-protons of **1a** and **1b,** where the peak of α-protons are at 2.93 ppm and 2.58 ppm, respectively, as mentioned above, due to the lack of electron density of ethyl substituent to compensate for the cationic charge on the phosphorus atom.

### 3.3. X-ray Analysis

**Figure 2:**
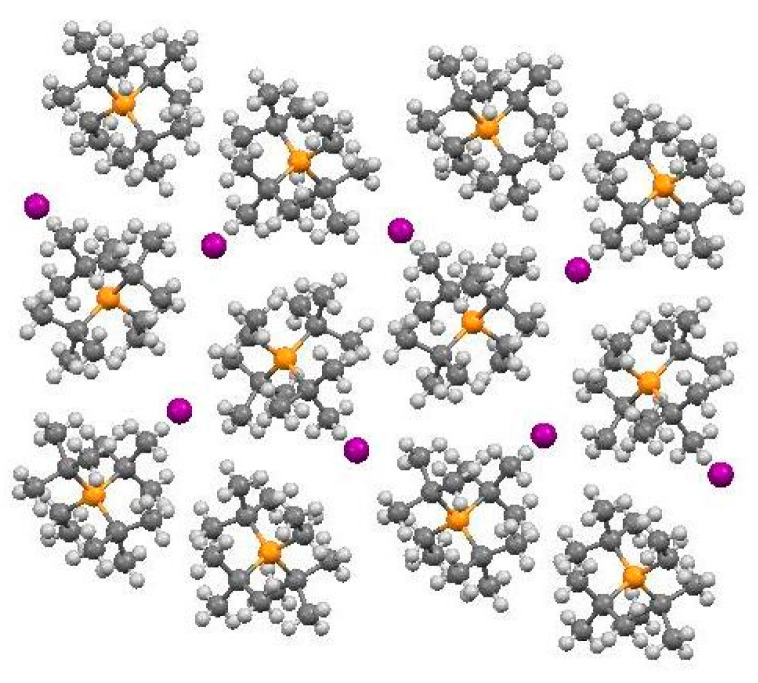
Crystal packing of **1a**.

**Figure 3:**
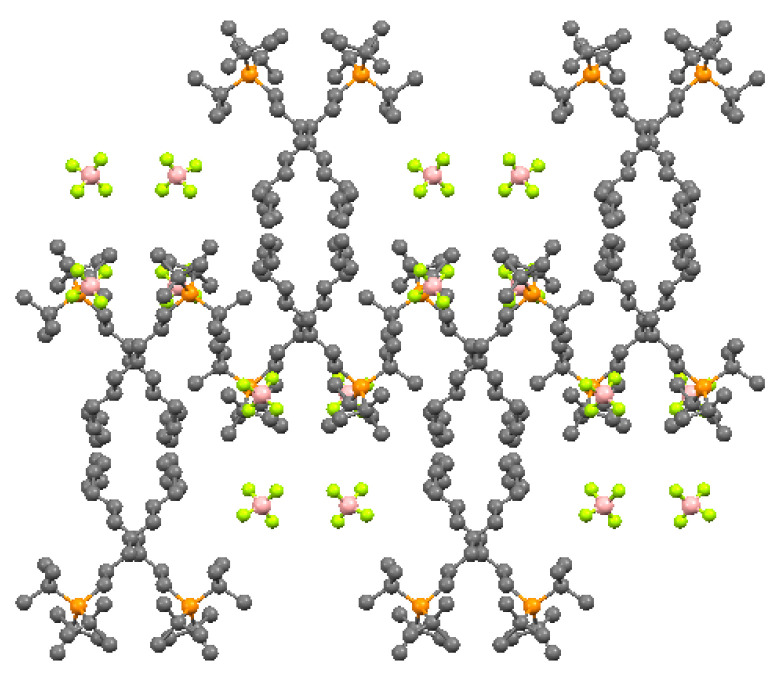
Crystal packing of **5b**.

**Figure 4:**
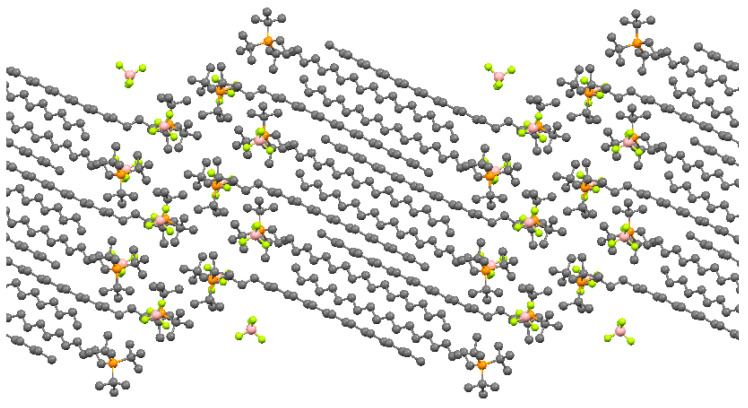
Crystal packing of **10b**.

Crystals suitable for X-ray structural analysis were obtained for two phosphonium salts **1a** and **10b**. Crystal packing of **5b** [56] was published earlier, and here it is compared with the new ones of **1a** and **10b** (Figure 2, Figure 3 and Figure 4).

**1a** possesses an ionic crystal lattice with cations and anions at the points indicated in Figure 2. The crystal packing is compact and ordered. 

Crystal packing of **5b** is more disordered than the previous one (Figure 3) [56]. Due to a linear alkyl substituent in the structure of phosphonium salt, the location of the molecules in the crystal is more complex.

The molecules of **10b** are arranged “head to tail” in the crystal (Figure 4). The long alkyl substituents are strictly parallel to each other. Hydrophilic (the head group) and hydrophobic (alkyl side-chain) regions are divided in the crystal packing.

Undoubtedly, the difference in the crystal packing of phosphonium salt affects their melting temperature. It should be noted that in the studied structures, anions are located at the hydrogen atom of the α-carbon atom of the *n*-alkyl substituent.

### 3.4. Melting Point

**Figure 5:**
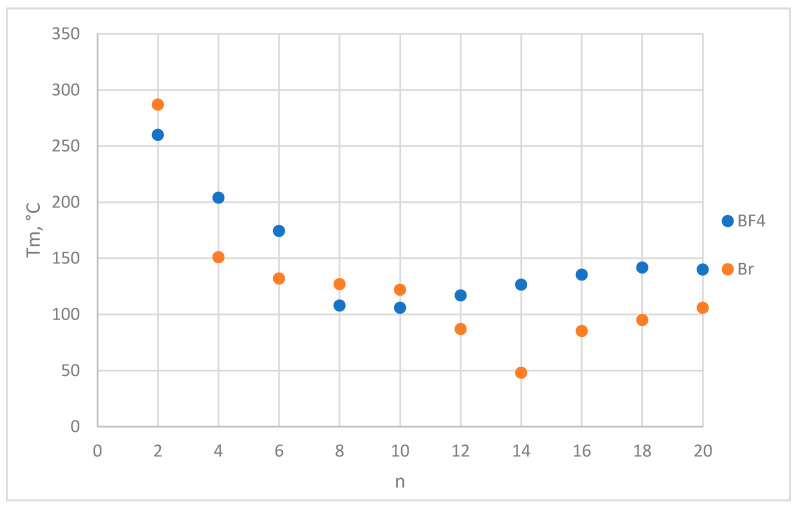
Melting points as a function of the number of carbons, *n*, in the fourth alkyl substituent on the phosphorus atom.

The melting temperatures (T_m_) of the synthesized phosphonium salts were measured. The trend of T_m_ as a function of the number of carbon atoms in the *n*-alkyl substitution on the phosphonium cation is presented in Figure 5. 

The melting points of the phosphonium salts reflected the type of crystal packaging, as expected. The first members of the series had high melting points above 200 °C. With an increase in the length of the alkyl chain, the symmetry of the molecules decreased, as well as the ordering of the crystal packing, which led to a drop in T_m_. The long alkyl substituents up to 20 carbon atoms were oriented strictly parallel to each other in the crystal packing, leading to the slight growth of the melting point.

A similar discussion has been previously reported on the number of 1-alkyl-3-methylimidazolium tetrafluoroborates [68]. The general trend was a decrease in melting points from methyl to *n*-octyl chains, followed by an increase in melting points and formation of a smectic liquid crystal phase with an increasing number of carbon atoms in *n*-alkyl substituent.

The melting points of phosphonium bromides in most cases are lower than those of phosphonium tetrafluoroborates. The presence of multiple hydrogen bonds C–H···F leads to a gain in intermolecular interaction and as a consequence an increase in the melting temperature.

### 3.5. PdNP Stabilization and TEM Sample Preparation

PdNPs proved to be superior catalysts in organic chemistry especially due to their “cocktail-type” behavior for cross-coupling reactions. However, thermodynamically unstable nanoparticles are susceptible to aggregation and require the presence of a stabilizing agent. Some organic salts were shown to be excellent stabilizers for metal nanoparticles. The fine-tuning of the organic salt structure allows one to vary the NP size and consequently its catalytic activity [69]. There are several articles devoted to the relationship between the nature of the stabilizer and the NP size. These studies have reported the influence of the cation structure [70], the anion volume [71] as well as both of them together [72]. Earlier, we have shown that steric hindrance of the phosphonium salt influences PdNPs’ catalytic activity [73].

For the present study, we chose palladium acetate as the source of nanoparticles. The procedure of PdNP formation represents the dissolution of palladium salt and corresponding phosphonium salt in the organic solvent. We tested two techniques of nanoparticles deposition on the grid: a “nanofishing” technique and the droplet technique. Pd(OAc)_2_ and **9b** were dissolved in ethyl alcohol. The sample for TEM measurement was prepared after 20 min of stirring of the solution.

“Nanofishing” is a special technique developed for studying liquid-phase colloidal systems and dispersed nanoparticles [74]. The procedure includes immersing a copper grid into the studied solution for 30–40 s and then rinsing the grid in acetone to remove excess organic components and drying the sample in the air.

The droplet technique is a standard approach including the application of 2–3 small drops of a studied solution containing nanoparticles onto a copper grid. After rinsing in acetone, the grid was dried in air and investigated by TEM.

**Figure 6:**
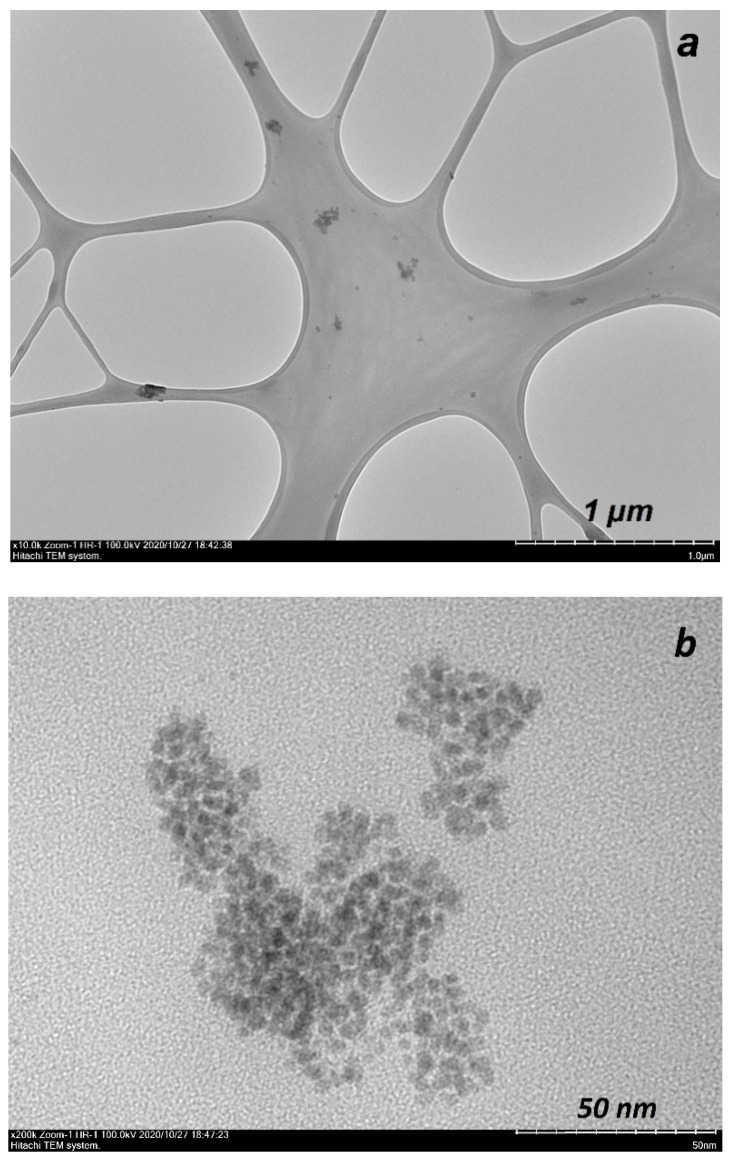

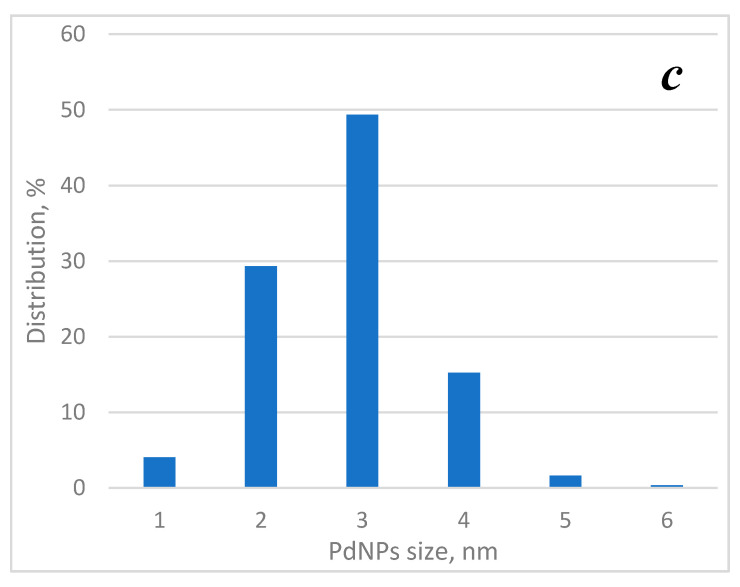
TEM of PdNPs using the “nanofishing” technique (**a**,**b**) and size distribution graph (**c**).

The solution of colloidal palladium prepared using **9b** was applied to compare these two methods. The sample prepared using the “nanofishing” technique showed that the agglomerates of PdNPs are small and well dispersed over the copper grid (Figure 6a,b). The average size of individual PdNPs was 2.81 ± 0.80 nm.

**Figure 7:**
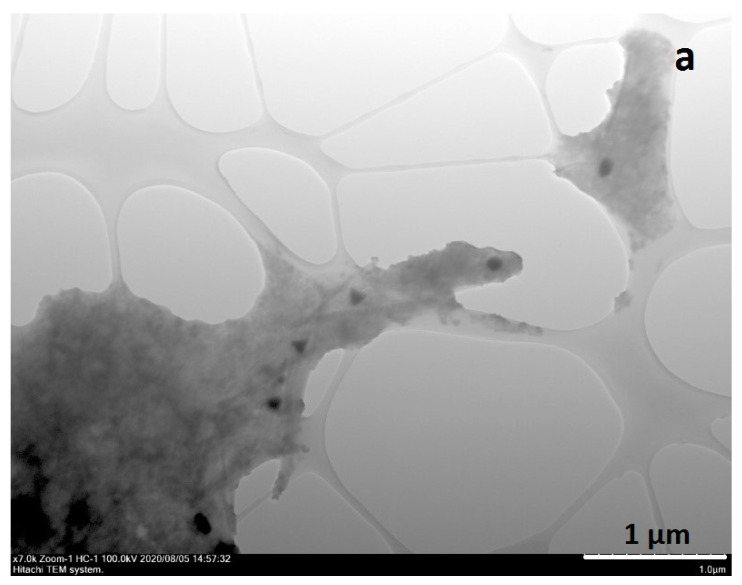

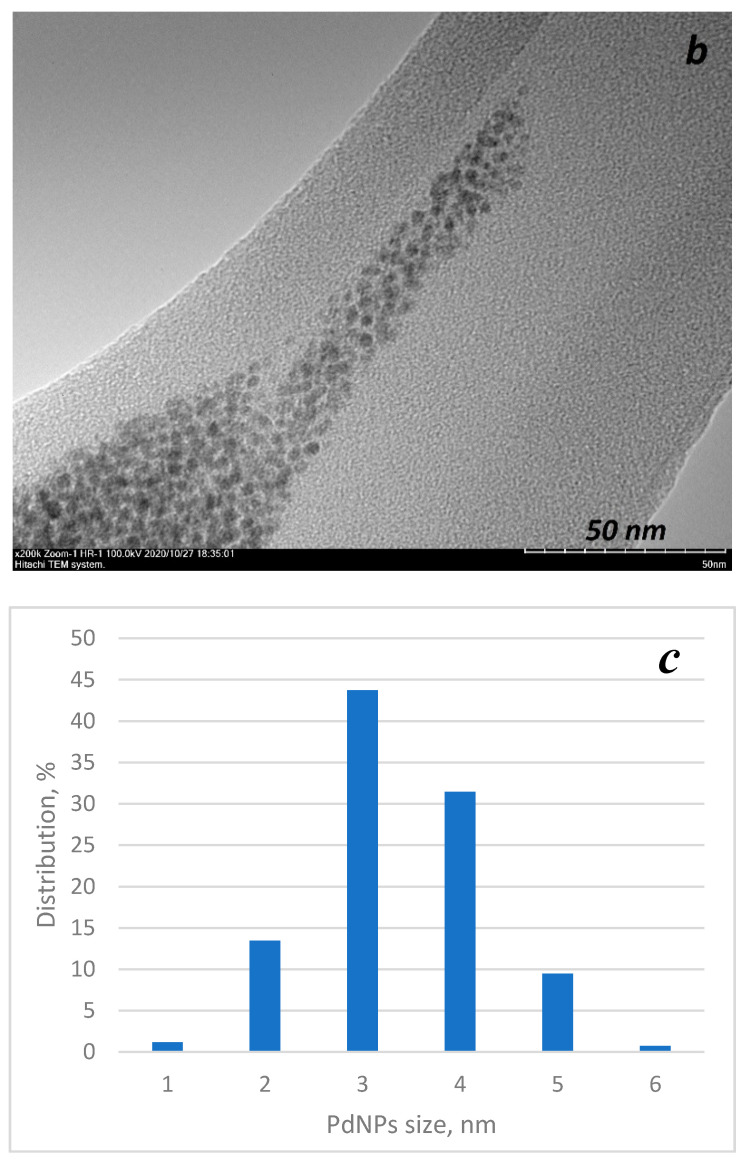
TEM of PdNPs using the droplet technique (**a**,**b**) and size distribution graph (**c**).

When the droplet technique was used, the sample contained the agglomerates of PdNPs that were located in a thick massive layer of the phosphonium salt (Figure 7a). These kinds of samples were opaque and hardly suitable for the TEM analysis. The average size of an individual nanoparticle was 3.37 ± 0.85 nm, which is close to the results of the “nanofishing” technique. It should be noted that individual nanoparticles were much more difficult to locate, and the fraction available for analysis was smaller than for the “nanofishing” procedure.

Thus, the “nanofishing” technique was chosen for the TEM samples preparation as the most optimal when phosphonium salt acts as a stabilizer.

Acetonitrile, chloroform, and ethanol were tested as media for the formation of PdNPs. Palladium acetate and **6b** were dissolved in 9 mL of each solvent. The sample for TEM measurement was prepared using the “nanofishing” technique after 20 min of stirring the solution.

**Figure 8:**
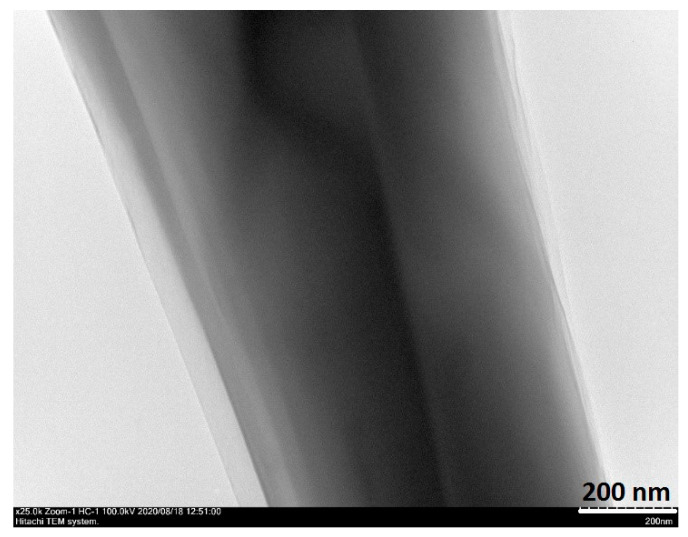
TEM sample prepared using the “nanofishing” procedure: Pd (OAc)_2_ and **6b** in acetonitrile.

**Figure 9:**
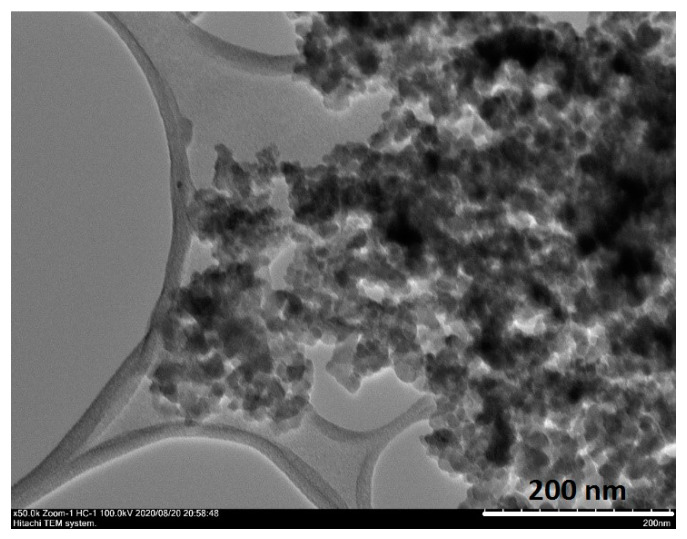
TEM samples prepared using the “nano fishing” procedure: Pd (OAc)_2_ and **6b** in chloroform.

We did not observe the formation of single PdNPs in acetonitrile and chloroform (Figure 8 and Figure 9). According to the literature, both chloroform [75] and acetonitrile [76] can solvate Pd (OAc)_2_ well in its tri-nuclear form without decomposition. While no particles were observed in acetonitrile (Figure 8), the agglomerates of particles were detected in chloroform (Figure 9).

**Figure 10:**
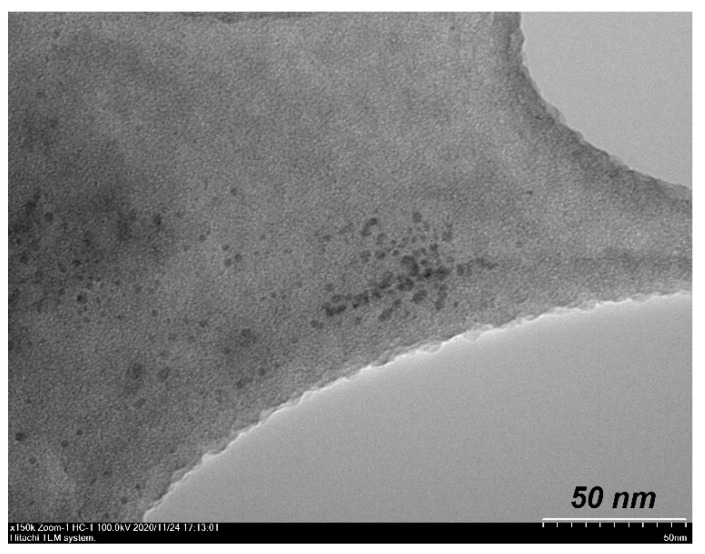
TEM samples prepared using the “nanofishing” procedure: Pd (OAc)_2_ and **6b** in ethanol.

Ethanol proved to promote the formation and stabilization of the PdNPs with **6b** (Figure 10), and the average size was 2.01 ± 0.72 nm. The PdNPs were located in large groups and possessed a uniform size. Individual nanoparticles, as well as the aggregates and molecules of phosphonium salt, were available in the ethanol solution, and it was of interest to study these systems in more detail. 

**Figure 11:**
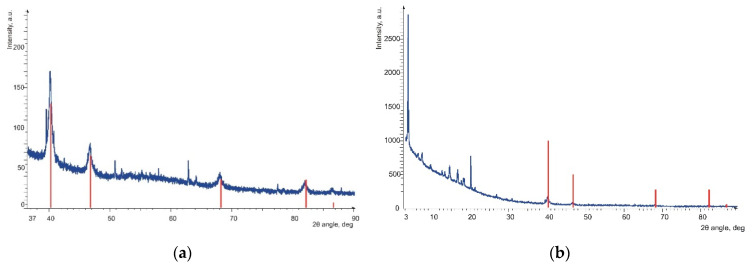
Experimental powder diffraction pattern of a sample PdNPs and **5b** in ethanol (**a**); enlarged part of the diffractogram (**b**). Red vertical bars show the positions of the interference peaks corresponding to the crystalline form of palladium Pd, syn., Code No. 00-001-1201.

The sample of the PdNPs and **5b** in ethanol was analyzed with X-ray powder diffraction (XRPD) method (Figure 11). According to XRPD data, the sample is a multicomponent system consisting of amorphous and several crystalline phases, the presence of which is evidenced by the pronounced difference in the half-width of several reflections. The search for suitable experimental X-ray diffraction patterns for identification in the PDF-2 database led to a suitable substance—the crystalline form of palladium Pd, syn., Code No. 00-001-1201—whose diffraction peak positions are shown in the figure as vertical bars. The diffuse nature of most of the palladium diffraction peaks indicates its nanostructure. In addition, a comparison was made between the experimental and calculated XRD-spectra (Figure S1 in Supplementary Materials).

Other possible crystalline components present in the test sample include the used salt and the products of its interaction or co-crystallization with the solvent. These were not analyzed at this stage of the research.

The quantitative assessment of the sizes of palladium Pd (0) crystallites from powder X-ray diffraction data was carried out based on analysis of the profiles of diffraction reflections corresponding to the crystalline form of palladium for the given sample. The crystallite sizes obtained from the profiles of diffraction lines corresponding to different families of planes are somewhat different but are close to each other, indicating the absence of noticeable anisometry of palladium crystallites. In general, their sizes are in the range of 8.8–17.8 nm (Table S1 in Supplementary Materials).

Additionally, the analysis of the size and shape of the obtained PdNPs in EtOH solutions of **10b** and **5b** salts was performed by small angle X-ray scattering (SAXS). According to SAXS data, both samples are characterized as heterogeneous, with the presence of the randomly oriented particles in solutions with dimensional characteristics in the range of 20–28 nm (Figures S2 and S3, Table S2 in Supplementary Materials). The increased particle sizes compared with the data of powder diffraction may indicate aggregation or a more complex morphology of the resulting nanoparticles, in particular, the formation of double layers on their surface.

### 3.6. Catalytic Suzuki Cross-Coupling Reaction

Earlier, we reported that PdNPs stabilized with the sterically hindered phosphonium salts were the effective catalyst in Suzuki [77] and Sonogashira [78] cross-coupling reactions. The wide scope of substrates involved in the reaction resulted in high conversion, e.g., chloroarenes, and successful recycling of the catalytic system was achieved [60].

**Scheme 3:**
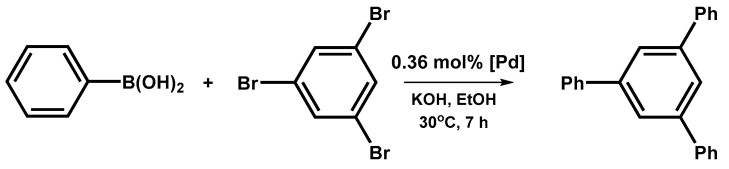
Suzuki cross-coupling reaction used as a model reaction.

The cross-coupling reaction of 1,3,5-tribromobenzene and phenylboronic acid (Scheme 3) was carried out using in situ prepared PdNPs under mild conditions (30 °C, 0.36 mol% [Pd], 7 h). Mainly tri-substituted benzene was formed as a product; the products of mono- and di-substitution were detected in the amounts 0–1.55% and 1.9–8.24% consequently. The PdNPs showed high catalytic activity and considerable stability. No “Pd black” precipitation was observed in the system before or after the reaction. It should be pointed out that a challenging model reaction was used in this study, where three C–C bonds should be made in a one-pot manner under mild conditions. Below, we describe the results of cross-coupling reaction for particular studied metal nanoparticles. 

### 3.7. PdNP Size Dynamics during the Cross-Coupling Reaction

The TEM sample preparation of the PdNPs stabilized by the sterically hindered phosphonium salt was carried out immediately after the preparation of the catalytic system (20 min of stirring the phosphonium salt and palladium acetate) and 7 h after the onset of Suzuki reaction using “nanofishing” techniques. For each phosphonium salt, 100–500 nanoparticles were processed to calculate the average size and standard deviation (Table 2).

The average size of the PdNPs before the Suzuki reaction is in a narrow range of 2.0–3.7 nm (except **1b**). Despite the difference in the structures of phosphonium salt and a strong difference in their properties, they all show the ability to stabilize very small nanoparticles, and consequently, the catalyst has a large active surface area. The standard deviation is only 0.7–1.8 nm, which indicates a high uniformity of nanoparticles in the solution.

**1b** stabilizes PdNPs 2–3 times larger and with a wider distribution interval. Some large PdNPs (up to 15 nm) were observed when **2b** was used as a stabilizer. This is perhaps associated with the high ionicity of these compounds that do not provide strong stabilization of nanoparticles, so the Pd atoms aggregate in bigger frameworks than in the presence of other phosphonium salts. 

Nanoparticles were not found in the solution containing **3b**. The experiment was repeated twice with the same result. The highly dynamic nature of stabilized metal clusters in the liquid phase may prevent trapping from the solution and detection by microscopy.

After Suzuki reaction, the size of PdNPs and the standard deviation increased insignificantly (Table 2). This indicates the high stability of the formed nanoparticles during the reaction and the efficiency of the phosphonium salts as stabilizing agents.

In the solution containing **3b,** the nanoparticles were detected after the catalytic reaction. The PdNPs were probably formed in the process of Suzuki reaction or when dynamic properties of the system were changed, which allowed their detection.

Interestingly, the PdNPs were not found in the presence of **4b** after the Suzuki reaction, which may indicate their agglomeration/precipitation or a change toward molecular stabilization with inefficient trapping from solution. Since the “palladium black” was not detected during the experiment the former case is unlikely. 

XRPD and SAXS analysis of the system of the PdNPs and **5b** in ethanol were performed in the solution with doubled concentration. The observed particles were larger than those measured by TEM technique due to the agglomeration process.

**Figure 12:**
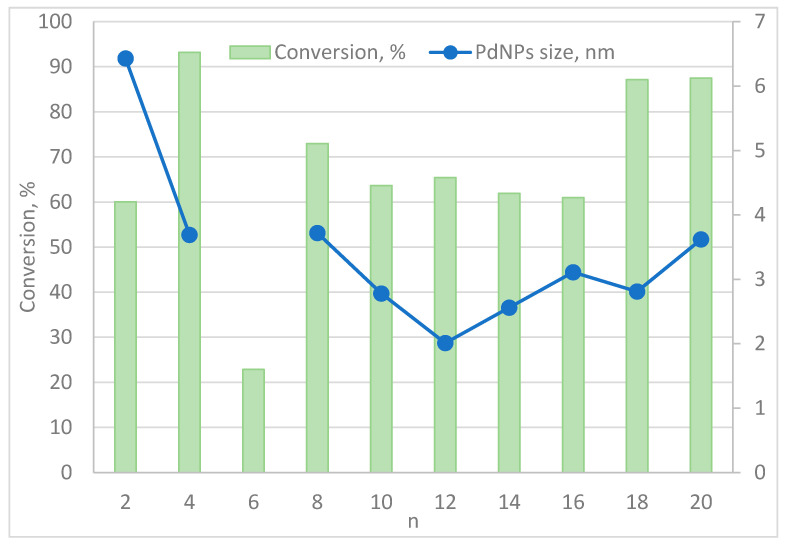
The PdNP size and the conversion of the Suzuki reaction depending on the phosphonium salt structure.

All the catalytic systems showed high catalytic activity in the studied Suzuki reaction (Figure 12). The conversion was in the range of 61–93% (excluding **3b**). The control experiment in the absence of the phosphonium salt was also carried out, for which the conversion was only around 6%.

The solution containing **3b** before the Suzuki reaction where PdNPs were not observed by “nanofishing” technique led to the lowest conversion (23%) (Table 2, Entry 3). 

Overall, the sizes of the PdNPs varied within the range of 2.0–6.4 nm, and the quantity of the surface Pd atoms in the corresponding nanoparticles may change significantly [79]. However, the conversion does not depend on this change. The results indicate that in the presence of phosphonium salt, catalytically active species can be generated from a large variety of PdNPs. 

The mechanism of PdNP-catalyzed cross-coupling reactions typically involves dynamic interconversion of catalytic species and leaching [80]. High catalytic efficiency observed for metal NPs of different sizes may be evidence for a dynamic catalytic system, where catalytically active centers can be formed in situ due to oxidative addition of the reagent and leaching [81,82,83]. This was previously discussed in the literature, including the discovery of “cocktail”-type catalytic systems for Pd-catalyzed cross-coupling reactions [80,84]. PdNPs were shown to play the role of convenient reservoirs of catalytically active species. In such cases, variations in the size of PdNPs did not significantly affect the overall performance of the reaction as soon as in situ generation of active centers became efficient. Dynamic “cocktail”-type catalytic systems have several practical advantages, such as good scope and easy practical usage. The present study shows that phosphonium salts possess excellent compatibility with PdNPs and facilitate the catalytic reaction.

The reasons for a difference in conversions should be sought in the environment of nanoparticles in the solution and the availability of their surface for the reactants. Since the stabilization of nanoparticles is carried out with the molecules of phosphonium salt and they are in the nearest surrounding, it is worth paying close attention to their properties in the solution. 

According to the literature, phosphonium salts have microheterogeneity [85], which is a special characteristic of the phosphonium salts containing the long alkyl substituents. Each molecule of such phosphonium salts is divided into hydrophobic and hydrophilic parts, which allows them to form aggregates in the solution. **9b** and **10b** form microreactors in which the substrates are concentrated around the nanoparticles. This leads to a higher conversion compared to phosphonium salts with medium-length alkyl chains (Figure 12). At the same time, TEM shows that the micelles/microreactors agglomerate into large aggregates consisting of hundreds to thousands of PdNPs surrounded by the molecules of phosphonium salt.

## 4. Conclusions

A series of novel phosphonium salts containing sterically hindered cations based on tri-*tert*-butylphosphine with varied *n*-alkyl substituent were synthesized, along with a detailed study of their ^1^H, ^13^C and ^31^P NMR, mass spectra, melting points and X-ray analysis.

The sterically hindered phosphonium salts support the formation of the PdNPs’ small average size with a narrow size distribution of 2.01 ± 0.72 to 6.43 ± 2.65 nm. The PdNPs demonstrate high catalytic activity in Suzuki cross-coupling. This is an important practical advantage, since variation in the side-chain length of phosphonium salts allows one to control their physical properties (for example, solubility in polar or non-polar solvents), but the NPs’ stabilization effect will remain. In such a case, this class of compounds may serve as universal NP stabilizers with tunable sidechain. 

The average size of PdNPs after the catalytic reaction slightly enhances the point about their excellent stability. The high catalytic efficiency observed for metallic NPs, regardless of their size within the range of ~2–6 nm, indicates a dynamic catalytic system. Catalytically active sites can be formed in different processes of catalyst evolution, where PdNPs play the role of a convenient reservoir of catalytically active species. In this case, variation in the size of the PdNPs does not significantly affect the overall catalytic performance. The wide scope of the applicable reactions and convenient practical usage make the application of dynamic “cocktail”-type catalytic systems advantageous. 

## Figures and Tables

**Table 1:** ^1^H NMR chemical shifts (δ (ppm), CDCl_3_, 400 MHz, 20 °C) and ^31^P{^1^H} NMR shifts (δ (ppm), CDCl_3_, 161.7 MHz, 20 °C).

Phosphonium Salt			Chemical Shift δ of α-Protons ^1^H NMR, ppm	Chemical Shift δ ^31^P NMR, ppm
N	Cation	Anion		
**1a**	t-Bu_3_P^+^C_2_H_5_	I^−^	2.93	50.53
**1b**		BF_4_^−^	2.58	49.96
**2a**	t-Bu_3_P^+^C_4_H_9_	Br^−^	2.65	49.54
**2b**		BF_4_^−^	2.31	49.17
**3a**	t-Bu_3_P^+^C_6_H_13_	Br^−^	2.61	49.51
**3b**		BF_4_^−^	2.32	49.16
**4a**	t-Bu_3_P^+^C_8_H_17_	Br^−^	2.55	49.30
**4b**		BF_4_^−^	2.29	49.08
**5a**	t-Bu_3_P^+^C_10_H_21_	Br^−^	2.56	49.78
**5b**		BF_4_^−^	2.29	49.08
**6a**	t-Bu_3_P^+^C_12_H_25_	Br^−^	2.59	50.06
**6b**		BF_4_^−^	2.27	49.03
**7a**	t-Bu_3_P^+^C_14_H_29_	Br^−^	2.68	50.01
**7b**		BF_4_^−^	2.27	49.08
**8a**	t-Bu_3_P^+^C_16_H_33_	Br^−^	2.65	49.53
**8b**		BF_4_^−^	2.29	49.07
**9a**	t-Bu_3_P^+^C_18_H_37_	Br^−^	2.62	50.12
**9b**		BF_4_^−^	2.29	49.08
**10a**	t-Bu_3_P^+^C_20_H_41_	Br^−^	2.54	49.51
**10b**		BF_4_^−^	2.33	49.11

**Table 2:** The average size of PdNPs before and after Suzuki reaction.

Entry	Phosphonium Salt	The Average Size of PdNPs, nm	The Average Size of PdNPs after Suzuki Reaction, nm
**1**	**1b**	6.43 ± 2.65	5.96 ± 2.08
**2**	**2b**	3.69 ± 1.35	4.25 ± 2.21
**3**	**3b**	not observed	3.57 ± 2.35
**4**	**4b**	3.72 ± 1.13	not observed
**5**	**5b**	2.78 ± 0.87	4.23 ± 1.36
**6**	**6b**	2.01 ± 0.72	3.57 ± 1.62
**7**	**7b**	2.56 ± 0.73	2.67 ± 0.83
**8**	**8b**	3.11 ± 1.08	3.59 ± 1.27
**9**	**9b**	2.81 ± 0.80	2.15 ± 0.66
**10**	**10b**	3.62 ± 1.57	4.59 ± 2.46

## Supplementary Materials

The following are available online at https://www.mdpi.com/2079-4991/10/12/2457/s1, the synthetic procedure and characterization of each phosphonium salt, Crystal data, X-ray Powder diffraction and Small angle X-ray scattering, Figure S1: Experimental diffraction pattern of Pd@5b sample and theoretical calculated curve, Figure S2: Screenshots of the 2D small-angle scattering pattern of the Pd@10b sample and SAXS diffraction intensity profiles (Intensity vs. s) at 23 °C, Table S1: The sizes of palladium crystallites, calculated from the parameters of diffraction peaks, for Pd@5b sample, Table S2: Calculated parameters for the samples Pd@10b and Pd@5b, Figure S3: The fitting of experimental SAXS curve for samples Pd@5b and Pd@10b in the case of Guinier-Porod model.
