# Supplementary material for: Sterically Hindered Phosphonium Salts: Structure, Properties and Palladium Nanoparticle Stabilization
Source: Nanomaterials (Basel). 2020 Dec 9;10(12):2457. doi: 10.3390/nano10122457 (PMC7763823; doi:10.3390/nano10122457)
Supplement: Supplementary file 1 [file nanomaterials-10-02457-s001.pdf]

Supplementary Materials

The synthesis of the phosphonium salts

Tri-*tert*-butyl(ethyl)phosphonium iodide (1a)

0.85 ml (10.63 mmol) iodoethane was added to 2.15 g (10.63 mmol) tri-*tert*-butylphosphine at 0°C. The mixture was allowed to reach room temperature. Formed white solid was washed with diethyl ether (3 X 10 ml); the residue of solvent was removed *in vacuo*.

White crystalline solid: 3.32 g, 87.14%; decomp. 285.0 °C; ^{1}H NMR ($CDCl_3$, 400 MHz) δ 2.93 (dq, $^{2}J_{PH}$ = 11.73 Hz, $^{3}J_{HH}$ = 7.43 Hz, 2H, P-CH_2), 1.71 (d, $^{3}J_{PH}$ = 13.76 Hz, 27H, P(C(CH_3)$_3$)$_3$), 1.67 (dt, $^{3}J_{PH}$ = 14.89 Hz, $^{3}J_{HH}$ = 7.46 Hz, 3H, P-CH_2-CH_3). ^{13}C NMR ($CDCl_3$, 100.6 MHz) δ 39.38 (d, $^{1}J_{PC}$ = 29.61 Hz, P(*C*(CH_3)$_3$)$_3$), 30.16 (s, P(C(CH_3)$_3$)$_3$, 13.20 (d, $^{1}J_{PC}$ = 37.38 Hz, P-CH_2-CH_3), 10.18 (d, $^{2}J_{PC}$ = 6.80 Hz, P-CH_2-CH_3). ^{31}P NMR ($CDCl_3$, (161.7 MHz) δ 50.53 (s). *m*/*z*: 231.2 ($C_{14}H_{32}P^+$, 100%).

Tri-*tert*-butyl(ethyl)phosphonium tetrafluoroborate (1b)

1.53 g (13.96 mmol) $NaBF_4$ was added to the solution of 2.50 g (6.98 mmol) tri-*tert*-butyl(ethyl)phosphonium iodide in 20 ml H_2O. White precipitate was formed, it was filtered, dissolved in CH_2Cl_2 and dried over $MgSO_4$ overnight. Then solution was filtered and dried *in vacuo*.

White solid: 1.67 g, 75.23%; decomp. 255.5 °C; ^{1}H NMR ($CDCl_3$, 400 MHz) δ 2.58 (dq, $^{2}J_{PH}$ = 11.96 Hz, $^{3}J_{HH}$ = 7.43 Hz, 2H, P-CH_2), 1.67 (d, $^{3}J_{PH}$ = 13.76 Hz, 27H, P(C(CH_3)$_3$)$_3$), 1.66 (dt, $^{3}J_{PH}$ = 14.90 Hz, $^{3}J_{HH}$ = 7.43 Hz, 3H, P-CH_2-CH_3). ^{13}C NMR ($CDCl_3$, 100.6 MHz) δ 39.25 (d, $^{1}J_{PC}$ = 29.60 Hz, P(*C*(CH_3)$_3$)$_3$), 29.79 (s, P(C(CH_3)$_3$)$_3$, 11.98 (d, $^{1}J_{PC}$ = 38.18 Hz, P-CH_2-CH_3), 9.75 (d, $^{2}J_{PC}$ = 7.06 Hz, P-CH_2-CH_3). ^{31}P NMR ($CDCl_3$, (161.7 MHz) δ 49.96 (s). *m*/*z*: 231.2 ($C_{14}H_{32}P^+$, 100%).

Tri-*tert*-butyl(butyl)phosphonium bromide (2a)

The mixture of 2.34 g (12.00 mmol) tri-*tert*-butylphosphine and 1.29 ml (12.00 mmol) 1-bromobutane was stirred for 6 hours at 100°C. The reaction mixture was washed with diethyl ether (3 X 10 ml) and dried *in vacuo*.

White powder: 2.58 g, 63.20%; mp. 151.0 °C; ^{1}H NMR ($CDCl_3$, 400 MHz) δ 2.65 (m, 2H, P-CH_2), 1.93 (m, 2H, P-CH_2-CH_2), 1.73 (m, $^{3}J_{HH}$ = 7.54 Hz, 2H, CH_2-CH_3), 1.71 (d, $^{3}J_{PH}$ = 13.87 Hz, 27H, P(C(CH_3)$_3$)$_3$), 1.02 (t, $^{3}J_{HH}$ = 7.29 Hz, 3H, CH_2-CH_3). ^{13}C NMR ($CDCl_3$, 100.6 MHz) δ 39.34 (d, $^{1}J_{PC}$ = 29.25 Hz, P(*C*(CH_3)$_3$)$_3$), 30.10 (s, P(C(CH_3)$_3$)$_3$), 27.18 (d, $^{3}J_{PC}$ = 6.44 Hz, CH_2-CH_3), 25.02 (d, $^{2}J_{PC}$ = 13.25 Hz, P-CH_2-CH_2), 18.77 (d, $^{1}J_{PC}$ = 34.98 Hz, P-CH_2), 13.79 (d, $^{4}J_{PC}$ = 0.74 Hz, CH_2-CH_3). ^{31}P NMR ($CDCl_3$, (161.7 MHz) δ 49.54 (s). *m*/*z*: 259.3 ($C_{16}H_{36}P^+$, 100%).

Tri-*tert*-butyl(butyl)phosphonium tetrafluoroborate (2b)

1.12 g (10.20 mmol) $NaBF_4$ was added to the solution of 1.73 g (5.10 mmol) tri-*tert*-butyl(butyl)phosphonium bromide in 10 ml H_2O. White precipitate was formed, it was filtered, dissolved in CH_2Cl_2 and dried over $MgSO_4$ overnight. Then solution was filtered and dried *in vacuo*.

White powder: 0.97 g, 54.80%; mp. 204.0 °C; ^{1}H NMR ($CDCl_3$, 400 MHz) δ 2.31 (m, 2H, P-CH_2), 1.90 (m, 2H, P-CH_2-CH_2), 1.65 (d, $^{3}J_{PH}$ = 13.96 Hz, 27H, P(C(CH_3)$_3$)$_3$), 1.63 (m, $^{3}J_{HH}$ = 7.56 Hz, 2H, CH_2-CH_3), 1.00 (t, $^{3}J_{HH}$ = 7.35 Hz, 3H, CH_2-CH_3). ^{13}C NMR ($CDCl_3$, 100.6 MHz) δ 39.27 (d, $^{1}J_{PC}$ = 29.42 Hz, P(*C*(CH_3)$_3$)$_3$), 29.80 (s, P(C(CH_3)$_3$)$_3$), 26.96 (d, $^{3}J_{PC}$ = 5.63 Hz, CH_2-CH_3), 24.76 (d, $^{2}J_{PC}$ = 13.14 Hz, P-CH_2-CH_2), 18.24 (d, $^{1}J_{PC}$ = 35.55 Hz, P-CH_2), 13.60 (d, CH_2-CH_3). ^{31}P NMR ($CDCl_3$, (161.7 MHz) δ 49.17 (s). *m*/*z*: 259.3 ($C_{16}H_{36}P^+$, 100%).

Tri-*tert*-butyl(hexyl)phosphonium bromide (3a)

The mixture of 2.357 g (11.65 mmol) tri-*tert*-butylphosphine and 1,64 ml (11,65 mmol) 1-bromohexane was stirred for 6 hours at 110°C. After cooling the reaction mixture was washed with diethyl ether (3 X 10 ml) and dried *in vacuo*.

White powder: 3.601 g, 84.12%; mp. 132°C; ^{1}H NMR ($CDCl_3$, 400 MHz) δ 2.61 (m, 2H, P-CH_2), 1.93 (m, 2H, P-CH_2-CH_2), 1.71 (d, $^{3}J_{PH}$ = 13.87 Hz, 27H, P(C(CH_3)$_3$)$_3$), 1.69 (m, 2H, P-CH_2-CH_2-CH_2), 1.35 (м, 4H, CH_2-CH_2-CH_3), 0.91 (t, $^{3}J_{HH}$ = 7.04 Hz, 3H, CH_2-CH_3). ^{13}C NMR ($CDCl_3$, 100.6 MHz) δ 39.34 (d, $^{1}J_{PC}$ = 29.50 Hz, P(*C*(CH_3)$_3$)$_3$), 31.52 (d, $^{2}J_{PC}$ = 12.40 Hz, P-CH_2-CH_2), 31.38 (s, CH_2-CH_2-CH_3), 30.11 (s, P(C(CH_3)$_3$)$_3$), 25.16 (d,

$^{3}J_{PC}$ = 6.38 Hz, *C*H2-CH2-CH2-CH3), 22.37 (s, *C*H2-CH3), 19.04 (d, $^{1}J_{PC}$ = 34.46 Hz, P-CH2), 13.99 (c, CH2-*C*H3). ^{31}P NMR (CDCl3, (161.7 MHz) δ 49.51 (s). *m/z*: 287.3 ($C_{18}H_{40}P^{+}$, 100%).

Tri-*tert*-butyl(hexyl)phosphonium tetrafluoroborate (3b)

1.47 g (13.40 mmol) $NaBF_4$ was added to the solution of 2.462 g (6.70 mmol) tri-*tert*-butyl(hexyl)phosphonium bromide in 8 ml H_2O. White precipitate was formed, it was filtered, dissolved in CH_2Cl_2 and dried over $MgSO_4$ overnight. Then solution was filtered and dried *in vacuo*.

White powder: 2.131 g, 84.97%; mp. 174.4°C; ^{1}H NMR ($CDCl_3$, 400 MHz) δ 2.32 (m, 2H, P-C*H2*), 1.92 (m, 2H, P-CH2-C*H2*), 1.66 (d, $^{3}J_{PH}$ = 13.90 Hz, 27H, P(C(C*H3*)3)3), 1.63 (m, 2H, P-CH2-CH2-C*H2*), 1.36 (m, 4H), 0.93 (t, $^{3}J_{HH}$ = 6.98 Hz, 3H, CH2-C*H3*). ^{13}C NMR ($CDCl_3$, 100.6 MHz) δ 39.29 (d, $^{1}J_{PC}$ = 29.54 Hz, P(*C*(CH3)3)3), 31.34 (d, $^{2}J_{PC}$ = 12.48 Hz, P-CH2-*C*H2), 31.25 (s), 29.82 (s, P(C(*C*H3)3)3), 24.99 (d, $^{3}J_{PC}$ = 6.53 Hz, *C*H2-CH2-CH2-CH3), 22.39 (s, CH2), 18.53 (d, $^{1}J_{PC}$ = 35.21 Hz, P-CH2), 13.96 (s, CH2-*C*H3). ^{31}P NMR ($CDCl_3$, (161.7 MHz) δ 49.61 (s). *m/z*: 287.3 ($C_{18}H_{40}P^{+}$, 100%).

Tri-*tert*-butyl(octyl)phosphonium bromide (4a)

The mixture of 2.49 g (12.31 mmol) tri-*tert*-butylphosphine and 2.13 ml (12.31 mmol) 1-bromooctane was stirred for 5 hours at 90°C. The reaction mixture was washed with diethyl ether (3 X 15 ml) and dried *in vacuo*.

White powder: 4.50 g, 92.46%; 127.0 °C. ^{1}H NMR ($CDCl_3$, 400 MHz) δ 2.55 (m, 2H, P-C*H2*), 1.89 (m, 2H, P-CH2-C*H2*), 1.66 (d, $^{3}J_{PH}$ = 13.92 Hz, 27H, P(C(C*H3*)3)3), 1.63 (m, 2H, P-CH2-CH2-C*H2*), 1.37-1.21 (m, 8H, CH2), 0.85 (t, $^{3}J_{HH}$ = 6.67 Hz, 3H, CH2-C*H3*). ^{13}C NMR ($CDCl_3$, 100.6 MHz) δ 39.34 (d, $^{1}J_{PC}$ = 29.29 Hz, P(*C*(CH3)3)3), 31.86 (s, CH2), 31.69 (d, $^{2}J_{PC}$ = 12.81 Hz, P-CH2-*C*H2), 30.11 (s, P(C(*C*H3)3)3), 29.22 (s, CH2), 28.96 (s, P-CH2-CH2-CH2-*C*H2), 25.20 (d, $^{3}J_{PC}$ = 6.49 Hz, P-CH2-CH2-*C*H2), 22.54 (s, CH2), 19.04 (d, $^{1}J_{PC}$ = 34.40 Hz, P-CH2), 14.03 (s, CH2-*C*H3). ^{31}P NMR ($CDCl_3$, (161.7 MHz) δ 49.30 (s). *m/z*: 315.3 ($C_{20}H_{44}P^{+}$, 100%).

Tri-*tert*-butyl(octyl)phosphonium tetrafluoroborate (4b)

1.65 g (15.07 mmol) $NaBF_4$ was added to 2.98 g (7.54 mmol) tri-*tert*-butyl(octyl)phosphonium bromide dissolved in 15 ml H_2O. White precipitate was formed, filtered, dissolved in CH_2Cl_2 and dried over $MgSO_4$ overnight. Then solution was filtered and dried *in vacuo*.

White powder: 2.37 g, 78.20 %; mp. 108.0 °C. ^{1}H NMR ($CDCl_3$, 400 MHz) δ 2.29 (m, 2H, P-C*H2*), 1.91 (m, 2H, P-CH2-C*H2*), 1.65 (d, $^{3}J_{PH}$ = 13.94 Hz, 27H, P(C(C*H3*)3)3), 1.58 (m, 2H, P-CH2-CH2-C*H2*), 1.40-1.24 (m, 8H, CH2), 0.89 (t, $^{3}J_{HH}$ = 6.82 Hz, 3H, CH2-C*H3*). ^{13}C NMR ($CDCl_3$, 100.6 MHz) δ 39.32 (d, $^{1}J_{PC}$ = 29.22 Hz, P(*C*(CH3)3)3), 31.73 (s, CH2), 31.70 (d, $^{2}J_{PC}$ = 12.41 Hz, P-CH2-*C*H2), 29.86 (s, P(C(*C*H3)3)3), 29.13 (s, CH2), 29.01 (s, P-CH2-CH2-CH2-*C*H2), 25.07 (d, $^{3}J_{PC}$ = 5.86 Hz, P-CH2-CH2-*C*H2), 22.60 (s, CH2), 18.58 (d, $^{1}J_{PC}$ = 35.23 Hz, P-CH2), 14.07 (s, CH2-*C*H3). ^{31}P NMR ($CDCl_3$, (161.7 MHz) δ 49.08 (s). *m/z*: 315.4 ($C_{20}H_{44}P^{+}$, 100%).

Tri-*tert*-butyl(decyl)phosphonium bromide (5a)

The mixture of 1.78 g (8,80 mmol) tri-*tert*-butylphosphine and 1.83 ml (8,80 mmol) 1-bromodecane was stirred for 5 hours at 100°C. The reaction mixture was washed with diethyl ether (4 X 10 ml) and dried *in vacuo*.

White powder: 3.01 g, 83.38 %; mp.122.0 °C; ^{1}H NMR ($CDCl_3$, 400 MHz) δ 2.56 (m, 2H, P-C*H2*), 1.91 (m, 2H, P-CH2-C*H2*), 1.67 (d, $^{3}J_{PH}$ = Hz, 27H, P(C(C*H3*)3)3), 1.66 (m, 2H), 1.37-1.20 (m, 14H), 0.86 (t, $^{3}J_{HH}$ = 6.86 Hz, 3H, CH2-C*H3*). ^{13}C NMR ($CDCl_3$, 100.6 MHz) δ = 39.33 (d, $^{1}J_{PC}$ = 29.32 Hz, *C*(CH3)3), 31.90 (d, $^{2}J_{PC}$ = 12.69 Hz, P-CH2-*C*H2), 31.83 (s, CH2), 30.11 (s, C(*C*H3)3), 29.52 (s, CH2), 29.34 (s, CH2), 29.31 (s, CH2), 29.24 (s, CH2), 25.21 (d, $^{3}J_{PH}$ = 7.72 Hz, P-CH2-CH2-*C*H2), 22.64 (s, *C*H2-CH3), 19.02 (d, $^{1}J_{PC}$ = 34.45 Hz, P-CH2), 14.10 (c, CH2-*C*H3). ^{31}P NMR ($CDCl_3$, (161.7 MHz) δ 49.78 (s). *m/z*: 343.4 ($C_{22}H_{48}P^{+}$, 100%).

Tri-*tert*-butyl(decyl)phosphonium tetrafluoroborate (5b)

1.23 g (11,24 mmol) $NaBF_4$ was added to the solution of 2.38 g (5,62 mmol) tri-*tert*-butyl(decyl)phosphonium bromide in 15 ml. White precipitate was formed, filtered, dissolved in CH_2Cl_2 and dried over $MgSO_4$ overnight. Then solution was filtered and dried *in vacuo*.

White powder: 1.99 g, 82.23 %; mp. 106.0 °C; ^{1}H NMR ($CDCl_3$, 400 MHz) δ 2.47 (m, 2H, P-C*H2*), 1.85 (m, 2H, P-CH2-C*H2*), 1.62 (d, $^{3}J_{PH}$ = 13.69 Hz, 27H, P(C(C*H3*)3)3), 1.19 (m, 14H), 0.81 (t, $^{3}J_{HH}$ = 6.6 Hz, 3H, CH2-

CH_3). ^{13}C NMR (CDCl$_3$, 100.6 MHz) δ (CDCl$_3$) δ = 39.24 (d, $^1J_{PC}$ = 29.306 Hz, C(CH$_3$)$_3$), 31.84 (d, $^2J_{PC}$ = 12.84 Hz, P-CH$_2$-CH$_2$), 31.83 (s, CH$_2$), 29.89 (s, C(CH$_3$)$_3$), 29.49 (s, CH$_2$), 29.33 (s, CH$_2$), 29.23 (s, CH$_2$), 29.20 (s, CH$_2$), 25.08 (d, $^3J_{PH}$ = 6.56 Hz, P-CH$_2$-CH$_2$-CH$_2$), 22.62 (s, CH$_2$-CH$_3$), 18.50 (d, $^1J_{PC}$ = 35.07 Hz, P-CH$_2$), 14.11 (c, CH$_2$-CH$_3$). ^{31}P NMR (CDCl$_3$, (161.7 MHz) δ 49.08 (s). *m/z*: 343.4 ($C_{22}H_{48}P^+$, 100%).

Tri-*tert*-butyl(dodecyl)phosphonium bromide (6a)

The mixture of 1.81 g (8.95 mmol) tri-*tert*-butylphosphine and 2.14 ml (8.95 mmol) 1-bromododecane was stirred for 4 hours at 100°C. The reaction mixture was washed with diethyl ether and dried *in vacuo*.

White powder: 2.87 g, 71.04%; mp. 87.0 °C; ^{1}H NMR (CDCl$_3$, 400 MHz) δ 2.72 (m, 2H, P-CH_2), 1.94 (m, 2H, P-CH$_2$-CH_2), 1.71 (d, $^3J_{PH}$ = 13.90 Hz, 27H, P(C(CH_3)$_3$)$_3$), 1.40-1.25 (m, 18H), 0.90 (t, $^3J_{HH}$ = 6.81 Hz, 3H, CH$_2$-CH_3). ^{13}C NMR (CDCl$_3$, 100.6 MHz) δ 39.35 (d, $^1J_{PC}$ = 29.52 Hz, C(CH$_3$)$_3$), 31.89 (d, $^2J_{PC}$ = 12.45 Hz, P-CH$_2$-CH$_2$), 31.86 (s, CH$_2$), 30.12 (s, C(CH$_3$)$_3$), 29.68-29.49 (m, 3CH$_2$), 29.39-29.22 (m, 3CH$_2$), 25.22 (d, $^3J_{PC}$ = 6.62 Hz, P-CH$_2$-CH$_2$-CH$_2$), 22.64 (s, CH$_2$-CH$_3$), 19.06 (d, $^1J_{PC}$ = 34.16 Hz, P-CH$_2$), 14.08 (s, CH$_2$-CH$_3$). ^{31}P NMR (CDCl$_3$, (161.7 MHz) δ 49.7 (s). *m/z*: 371.4 ($C_{24}H_{52}P^+$, 100%).

Tri-*tert*-butyl(dodecyl)phosphonium tetrafluoroborate (6b)

0.99 g (9.0 mmol) $NaBF_4$ was added to the solution of 3.0 g (6.0 mmol) tri-*tert*-butyl(dodecyl)phosphonium bromide in 15 ml H_2O. White precipitate was formed, filtered, dissolved in CH_2Cl_2 and dried over $MgSO_4$ overnight. Then solution was filtered and dried *in vacuo*.

White crystalline powder: 1.9 g, 68.8%; mp. 117.0 °C. ^{1}H NMR (CDCl$_3$, 400 MHz) δ 2.39 (m, 2H, P-CH_2), 1.92 (m, 2H, P-CH$_2$-CH_2), 1.67 (d, $^3J_{PH}$ = 13.91 Hz, 27H, P(C(CH_3)$_3$)$_3$), 1.38-1.23 (m, 18H), 0.89 (t, $^3J_{HH}$ = 7.24 Hz, 3H, CH$_2$-CH_3). ^{13}C NMR (CDCl$_3$, 100.6 MHz) δ 39.75 (d, $^1J_{PC}$ = 29.34 Hz, C(CH$_3$)$_3$), 32.31 (s, CH$_2$), 32.20 (d, $^2J_{PC}$ = 12.65 Hz, P-CH$_2$-CH$_2$), 30.32 (s, C(CH$_3$)$_3$), 30.09 (s, CH$_2$), 30.02 (s, CH$_2$), 29.99 (s, CH$_2$), 29.78 (s, CH$_2$), 29.73 (s, CH$_2$), 29.62 (s, CH$_2$), 25.48 (d, $^3J_{PC}$ = 6.60 Hz, P-CH$_2$-CH$_2$-CH$_2$), 23.08 (s, CH$_2$-CH$_3$), 19.10 (d, $^1J_{PC}$ = 34.85 Hz, P-CH$_2$), 14.52 (s, CH$_2$-CH$_3$). ^{31}P NMR (CDCl$_3$, (161.7 MHz) δ 49.3 (s). *m/z*: 371.5 ($C_{24}H_{52}P^+$, 100%).

Tri-*tert*-butyl(tetradecyl)phosphonium bromide (7a)

The mixture of 3.00 g (14.8 mmol) tri-*tert*-butylphosphine and 4.4 ml (14.8 mmol) 1-bromotetradecane was stirred for 8 hours at 100°C. The reaction mixture was washed with diethyl ether (4 X 15 ml) and dried *in vacuo*.

White amorphous powder: 4.5 g, 63.4%; mp. 48 °C; ^{1}H NMR (CDCl$_3$, 400 MHz) δ 2.68 (m, 2H, P-CH_2), 1.93 (m, 2H, P-CH$_2$-CH_2), 1.70 (d, $^3J_{PH}$ = 13.80 Hz, 27H, P(C(CH_3)$_3$)$_3$), 1.40-1.22 (m, 22H), 0.89 (t, $^3J_{HH}$ = 6.90 Hz, 3H, CH$_2$-CH_3). ^{13}C NMR (CDCl$_3$, 100.6 MHz) δ 39.75 (d, $^1J_{PC}$ = 29.34 Hz, C(CH$_3$)$_3$), 32.35 (s, CH$_2$), 32.18 (d, $^2J_{PC}$ = 12.47 Hz, P-CH$_2$-CH$_2$), 30.32 (s, C(CH$_3$)$_3$), 30.17-29.97 (m, CH$_2$-(CH$_2$)$_2$-CH$_2$), 29.80 (s, CH$_2$), 29.78 (s, CH$_2$), 29.66 (s, CH$_2$), 25.53 (d, $^3J_{PC}$ = 6.60 Hz, P-CH$_2$-CH$_2$-CH$_2$), 23.11 (s, CH$_2$-CH$_3$), 19.03 (d, $^1J_{PC}$ = 34.85 Hz, P-CH$_2$), 14.53 (s, CH$_2$-CH$_3$). ^{31}P NMR (CDCl$_3$, (161.7 MHz) δ 50.3 (s). *m/z*: 399.64 ($C_{26}H_{56}P^+$, 100%).

Tri-*tert*-butyl(tetradecyl)phosphonium tetrafluoroborate (7b)

1.03 g (9.4 mmol) $NaBF_4$ was added to the solution of 3.0 g (6.3 mmol) tri-*tert*-butyl(tetradecyl)phosphonium bromide in 20 ml H_2O. White precipitate was formed, filtered, dissolved in CH_2Cl_2 and dried over $MgSO_4$ overnight. Then solution was filtered and dried *in vacuo*.

White crystalline powder: 2.01 g, 65.9%; mp. 126.5 °C; ^{1}H NMR (CDCl$_3$, 400 MHz) δ 2.27 (m, 2H, P-CH_2), 1.89 (m, 2H, P-CH$_2$-CH_2), 1.64 (d, $^3J_{PH}$ = 13.09 Hz, 27H, P(C(CH_3)$_3$)$_3$), 1.56 (m, 2H), 1.37-1.22 (m, 20H), 0.88 (t, $^3J_{HH}$ = 6.82 Hz, 3H, CH$_2$-CH_3). ^{13}C NMR (CDCl$_3$, 100.6 MHz) δ 39.28 (d, $^1J_{PC}$ = 29.38 Hz, C(CH$_3$)$_3$), 31.89 (s, CH$_2$), 31.70 (d, $^2J_{PC}$ = 12.33 Hz, P-CH$_2$-CH$_2$), 29.80 (s, C(CH$_3$)$_3$), 29.71-29.07 (m, 7CH$_2$), 25.03 (d, $^3J_{PC}$ = 6.52 Hz, P-CH$_2$-CH$_2$-CH$_2$), 22.65 (s, CH$_2$-CH$_3$), 18.53 (d, $^1J_{PC}$ = 34.75 Hz, P-CH$_2$), 14.08 (s, CH$_2$-CH$_3$). ^{31}P NMR (CDCl$_3$, (161.7 MHz) δ 49.3 (s). *m/z*: 399.4 ($C_{26}H_{56}P^+$, 100%).

Tri-*tert*-butyl(hexadecyl)phosphonium bromide (8a)

2.12 g (10.48 mmol) tri-*tert*-butylphosphine and 3.20 g (10.48 mmol) 1-bromohexadecane were dissolved in 10 ml acetonitrile. Reaction mixture was stirred for 10 hours at 80°C in inert atmosphere. Cooled mixture was evaporated and washed with diethyl ether (4 X 10 ml) and dried *in vacuo*.

White amorphous powder: 3.28 g, 61.65%; mp. 85.2 °C; ^{1}H NMR ($CDCl_3$, 400 MHz) δ 2.65 (m, 2H, P-C*H*$_2$), 1.93 (m, 2H, P-CH_2-C*H*$_2$), 1.70 (d, $^3J_{PH}$ = 13.90 Hz, 27H, P(C(C*H*$_3$)$_3$)$_3$), 1.70 (m, 2H), 1.40-1.23 (m, 24H), 0.89 (t, $^3J_{HH}$ = 6.83 Hz, 3H, CH_2-C*H*$_3$). ^{13}C NMR ($CDCl_3$, 100.6 MHz) δ 39.32 (d, $^1J_{PC}$ = 29.52 Hz, *C*(CH_3)$_3$), 31.88 (s, CH_2), 31.90 (d, $^2J_{PC}$ = 12.26 Hz, P-CH_2-CH_2), 30.08 (s, C(CH_3)$_3$), 29.75-29.53 (m, 5CH_2), 29.40-29.26 (m, 5CH_2), 25.18 (d, $^3J_{PC}$ = 6.57 Hz, P-CH_2-CH_2-CH_2), 22.65 (s, *C*H$_2$-CH_3), 18.99 (d, $^1J_{PC}$ = 34.98 Hz, P-CH_2), 14.10 (s, CH_2-*C*H$_3$). ^{31}P NMR ($CDCl_3$, (161.7 MHz) δ 49.53 (s). *m/z*: 427.5 ($C_{28}H_{60}P^+$, 100%).

Tri-*tert*-butyl(hexadecyl)phosphonium tetrafluoroborate (8b)

2,50 g (4.92 mmol) tri-*tert*-butyl(hexadecyl)phosphonium bromide was dissolved in 20 ml of water. 1.08 g (9.85 mmol) $NaBF_4$ was added to the solution. White precipitate was formed, it was filtered, dissolved in CH_2Cl_2 and dried over $MgSO_4$ overnight. Then solution was filtered and evaporated *in vacuo*.

White crystalline powder: 1.11 g, 40.22%; mp. 133.5°C; ^{1}H NMR ($CDCl_3$, 400 MHz) δ 2.29 (m, 2H, P-C*H*$_2$), 1.91 (m, 2H, P-CH_2-C*H*$_2$), 1.64 (d, $^3J_{PH}$ = 13.92 Hz, 27H, P(C(C*H*$_3$)$_3$)$_3$), 1.39-1.22 (m, 26H), 0.89 (t, $^3J_{HH}$ = 6.99 Hz, 3H, CH_2-C*H*$_3$). ^{13}C NMR ($CDCl_3$, 100.6 MHz) δ 39.24 (d, $^1J_{PC}$ = 29.40 Hz, *C*(CH_3)$_3$), 31.90 (s, CH_2), 31.71 (d, $^2J_{PC}$ = 12.59 Hz, P-CH_2-CH_2), 29.77 (s, C(CH_3)$_3$), 29.72-29.60 (m, 6CH_2), 29.58 (s, CH_2), 29.38 (s, CH_2), 29.34 (s, CH_2), 29.19 (s, CH_2), 25.02 (d, $^3J_{PC}$ = 6.59 Hz, P-CH_2-CH_2-CH_2), 22.67 (s, *C*H$_2$-CH_3), 18.49 (d, $^1J_{PC}$ = 34.58 Hz, P-CH_2), 14.12 (s, CH_2-*C*H$_3$). ^{31}P NMR ($CDCl_3$, (161.7 MHz) δ 49.07 (s). *m/z*: 427.5 ($C_{28}H_{60}P^+$, 100%).

Tri-*tert*-butyl(octadecyl)phosphonium bromide (9a)

The mixture of 2.22 g (10.97 mmol) tri-*tert*-butylphosphine and 3.66 g (10.97 mmol) 1-bromooctadecane were dissolved in 10 ml CH_3CN. Reaction mixture was stirred for 10 hours at 80°C in inert atmosphere. After cooling the reaction solvent was removed in vacuum. The solid was washed with diethyl ether (4 X 10 ml) and dried *in vacuo*.

White amorphous powder: 4.87 g, 82.82%; mp. 93.7°C; ^{1}H NMR ($CDCl_3$, 400 MHz) δ 2.62 (m, 2H, P-C*H*$_2$), 1.92 (m, 2H, P-CH_2-C*H*$_2$), 1.69 (d, $^3J_{PH}$ = 14.39 Hz, 27H, P(C(C*H*$_3$)$_3$)$_3$),1.69 (m, 2H), 1.38-1.22 (m, 28H), 0.88 (t, $^3J_{HH}$ = 6.58 Hz, 3H, CH_2-C*H*$_3$). ^{13}C NMR ($CDCl_3$, 100.6 MHz) δ 39.31 (d, $^1J_{PC}$ = 29.38 Hz, *C*(CH_3)$_3$), 31.89 (s, CH_2), 31.90 (d, $^2J_{PC}$ = 13.06 Hz, P-CH_2-*C*H$_2$), 30.09 (s, C(CH_3)$_3$), 29.71-29.54 (m, 6CH_2), 29.38-29.26 (m, 4CH_2), 25.20 (d, $^3J_{PC}$ = 6.96 Hz, P-CH_2-CH_2-CH_2), 22.66 (s, *C*H$_2$-CH_3), 19.00 (d, $^1J_{PC}$ = 33.93 Hz, P-CH_2), 14.11 (s, CH_2-CH_3). ^{31}P NMR ($CDCl_3$, (161.7 MHz) δ 50.12 (s). *m/z*: 455.5 ($C_{30}H_{64}P^+$, 100%).

Tri-*tert*-butyl(octadecyl)phosphonium tetrafluoroborate (9b)

3.50 g (6.53 mmol) of tri-*tert*-butyl(octadecyl)phosphonium bromide was dissolved in 35 ml of water. 1.43 g (13.07 mmol) $NaBF_4$ was added to the solution. White precipitate was formed, it was filtered, dissolved in CH_2Cl_2 and dried over $MgSO_4$ overnight. Then solution was filtered and evaporated *in vacuo*.

White powder: 3.25 g, 91.55%; mp. 141.8°C; ^{1}H NMR ($CDCl_3$, 400 MHz) δ 2.29 (m, 2H, P-C*H*$_2$), 1.91 (m, 2H, P-CH_2-C*H*$_2$), 1.65 (d, $^3J_{PH}$ = 13.89 Hz, 27H, P(C(C*H*$_3$)$_3$)$_3$), 1.58 (m, 2H), 1.39-1.22 (m, 28H), 0.90 (t, $^3J_{HH}$ = 6.85 Hz, 3H, CH_2-C*H*$_3$). ^{13}C NMR ($CDCl_3$, 100.6 MHz) δ 39.25 (d, $^1J_{PC}$ = 29.23 Hz, *C*(CH_3)$_3$), 31.91 (s, CH_2), 31.71 (d, $^2J_{PC}$ = 12.85 Hz, P-CH_2-CH_2), 29.77 (s, C(*C*H$_3$)$_3$), 29.74-29.61 (m, 8CH_2), 29.59 (s, CH_2), 29.38 (s, CH_2), 29.35 (s, CH_2), 29.19 (s, CH_2), 25.02 (d, $^3J_{PC}$ = 7.01 Hz, P-CH_2-CH_2-CH_2), 22.67 (s, *C*H$_2$-CH_3), 18.49 (d, $^1J_{PC}$ = 34.80 Hz, P-CH_2), 14.12 (s, CH_2-CH_3). ^{31}P NMR ($CDCl_3$, (161.7 MHz) δ 49.08 (s). *m/z*: 455.5 ($C_{30}H_{64}P^+$, 100%).

Tri-*tert*-butyl(eicosyl)phosphonium bromide (10a)

The mixture of 3.32 g (16.41 mmol) tri-*tert*-butylphosphine and 5.93 g (16.41 mmol) 1-bromoeicosane were dissolved in 20 ml CH_3CN. The mixture was stirred for 9 hours at 80°C in inert atmosphere. After cooling the reaction solvent was removed in vacuum. The solid was washed with diethyl ether (4 X 10 ml) and dried *in vacuo*.

White powder: 7.55 g, 81.60%; mp. 106.0 °C; ^{1}H NMR ($CDCl_3$, 400 MHz) δ 2.54 (m, 2H, P-C*H*$_2$), 1.89 (m, 2H, P-CH_2-C*H*$_2$), 1.66 (d, $^3J_{PH}$ = 13.42 Hz, 27H, P(C(C*H*$_3$)$_3$)$_3$), 1.69 (m, 2H), 1.35-1.18 (m, 32H), 0.89 (t, $^3J_{HH}$ = 6.60 Hz, 3H, CH_2-C*H*$_3$). ^{13}C NMR ($CDCl_3$, 100.6 MHz) δ 39.38 (d, $^1J_{PC}$ = 29.31 Hz, *C*(CH_3)$_3$), 31.90 (s, CH_2), 31.91 (d, $^2J_{PC}$ = 12.14 Hz, P-CH_2-CH_2), 30.14 (s, C(CH_3)$_3$), 29.78-29.55 (m, 10CH_2), 29.43-29.26 (m, 4CH_2), 25.23 (d, $^3J_{PC}$ = 6.62 Hz, P-CH_2-CH_2-CH_2), 22.65 (s, *C*H$_2$-CH_3), 19.08 (d, $^1J_{PC}$ = 34.86 Hz, P-CH_2), 14.08 (s, CH_2-CH_3). ^{31}P NMR ($CDCl_3$, (161.7 MHz) δ 49.51 (s). *m/z*: 483.6 ($C_{32}H_{68}P^+$, 100%).

Tri-*tert*-butyl(eicosyl)phosphonium tetrafluoroborate (10b)
5.60 g (9.93 mmol) of tri-*tert*-butyl(eicosyl)phosphonium bromide was dissolved in 60 ml of water. 2.18 g (19.87 mmol) $NaBF_4$ was added to the solution. White precipitate was formed, it was filtered, dissolved in CH_2Cl_2 and dried over $MgSO_4$ overnight. Then solution was filtered and evaporated *in vacuo*.
White powder: 4.70 g, 82.90%; mp. 140.0 °C; ^1H NMR ($CDCl_3$, 400 MHz) δ 2.33 (m, 2H, P-CH_2), 1.91 (m, 2H, P-CH_2-CH_2), 1.65 (d, $^3J_{PH}$ = 13.93 Hz, 27H, P(C(CH_3)$_3$)$_3$), 1.59 (m, 2H), 1.38-1.23 (m, 34H), 0.89 (t, $^3J_{HH}$ = 6.81 Hz, 3H, CH_2-CH_3). ^{13}C NMR ($CDCl_3$, 100.6 MHz) δ 39.73 (d, $^1J_{PC}$ = 29.60 Hz, *C*(CH_3)$_3$), 32.34 (s, CH_2), 32.14 (d, $^2J_{PC}$ = 12.47 Hz, P-CH_2-CH_2), 30.25 (s, C(CH_3)$_3$), 30.19-29.96 (m, 11CH_2), 29.81 (s, CH_2), 29.77 (s, CH_2), 29.64 (s, CH_2), 25.49 (d, $^3J_{PC}$ = 6.60 Hz, P-CH_2-CH_2-CH_2), 23.10 (s, *C*H_2-CH_3), 18.97 (d, $^1J_{PC}$ = 34.85 Hz, P-CH_2), 14.52 (s, CH_2-CH_3). ^{31}P NMR ($CDCl_3$, (161.7 MHz) δ 49.11 (s). *m/z*: 483.6 ($C_{32}H_{68}P^+$, 100%).

X-Ray data

Crystal Data for $C_{14}H_{32}IP$ (*M* =358.26 g/mol): monoclinic, space group P2$_1$/n (no. 14), *a* = 8.0828(16) Å, *b* = 13.954(3) Å, *c* = 15.054(3) Å, *β* = 95.559(4)°, *V* = 1689.9(6) Å^3, *Z* = 4, *T* = 150 K, μ(MoK*α*) = 1.970 mm^{-1}, *Dcalc* = 1.408 g/cm^3, 52985 reflections measured (3.988° ≤ 2Θ ≤ 63.34°), 5695 unique (R_{int} = 0.0234, R_{sigma} = 0.0116) which were used in all calculations. The final R_1 was 0.0140 (I > 2σ(I)) and wR_2 was 0.0379 (all data). CCDC refcode: 2027442.

Crystal Data for $C_{32}H_{68}BF_4P$ (*M* =570.64 g/mol): triclinic, space group P-1 (no. 2), *a* = 8.8804(6) Å, *b* = 11.4352(8) Å, *c* = 34.681(2) Å, *α* = 92.756(3)°, *β* = 92.254(3)°, *γ* = 92.955(3)°, *V* = 3510.0(4) Å^3, *Z* = 4, *T* = 150.15 K, μ(CuK*α*) = 1.007 mm^{-1}, *Dcalc* = 1.080 g/cm^3, 81452 reflections measured (5.106° ≤ 2Θ ≤ 137.658°), 12791 unique (R_{int} = 0.0426, R_{sigma} = 0.0285) which were used in all calculations. The final R_1 was 0.0482 (I > 2σ(I)) and wR_2 was 0.1288 (all data). CCDC refcode: 2031583.

Typical procedure for PdNPs preparation
0.0004 g (0.00178 mmol) of palladium acetate and 0.178 mmol of phosphonium salt (see Table below) was dissolved in 9 ml ethanol and stirred during 20 minutes at room temperature. The color of solution changes from transparent to light brownish grey.

Phosphonium salt	m, g
**1b**	0.057
**2b**	0.062
**3b**	0.067
**4b**	0.072
**5b**	0.077
**6b**	0.082
**7b**	0.087
**8b**	0.092
**9b**	0.097
**10b**	0.102

Procedure of PdNPs preparation for XRPD and SAXS (Pd@**5b)**
0.0008 g (0.00356 mmol) of palladium acetate and 0.154 g (0.356 mmol) of **5b** was dissolved in 9 ml ethanol and stirred during 20 minutes at room temperature.

Procedure of PdNPs preparation for SAXS (Pd@**10b)**
0.0008 g (0.00356 mmol) of palladium acetate and 0.204 g (0.356 mmol) of **10b** was dissolved in 9 ml ethanol and stirred during 20 minutes at room temperature.

X-ray powder diffraction

Sample Pd@**5b** in EtOH was applied in liquid form on the surface of a standard zero diffraction silicon plate, which reduces background scattering. After drying the layer, a few more layers were applied on top of it to increase the total amount of the sample. Patterns were recorded in the 2θ range between 3° and 95° in 0.008° steps with a step time of 1s. The sample was spun (15 rpm) throughout the data collection. For the sample four diffractograms were obtained, which were summed.

Processing of the obtained data performed using EVA[1] and TOPAS[2] software packages. The powder X-ray diffraction database PDF-2 (ICDD PDF-2, Release 2005–2009) was used to identify the crystalline phase. The crystallite size calculations were performed using the TOPAS software package[3] in several ways: the values, calculated from the half-width of the reflections (LVol-FWHM) and the integrated reflection intensity (LVol-IB), are the volume-weighted values of the crystallite sizes, and the CrySizeL parameter is the size of the crystallites in the direction perpendicular to the analyzed planes, with the Lorentz type of peak broadening. The minimization of the discrepancy between the experimental and calculated data in the refinement process was performed by the Rietveld method over the entire array of experimental data.

A full-profile analysis of the experimental diffraction data and refinement of the results by the Rietveld method were performed using the TOPAS software package, while crystalline palladium was analyzed as a separate phase, and the rest of the crystalline components of the sample were included to the second crystalline phase and were not analyzed in detail, the refinement results are shown in Figure S1. Calculations crystallite sizes are performed in several ways: the values, calculated from the half-width of the reflections (LVol-FWHM) and from the integrated intensity of the reflections (LVol-IB), are the volume-weighted average values of sizes of the crystallites, and the CrySizeL parameter is the crystallite size in the direction perpendicular to the analyzed planes, with the Lorentz-type broadening. The correctness of the comparison of the model (theoretical) and experimental curves was ensured by minimizing the convergence parameters R_{wp} and R_{exp}. The difference curve serves as an additional visual criterion for the convergence of the results, the deviation of which from the zero value could mean incomplete identification of the composition. The obtained results of the full-profile analysis with the determination of the crystallite sizes for the Pd@**5b** sample are shown in Table S1.

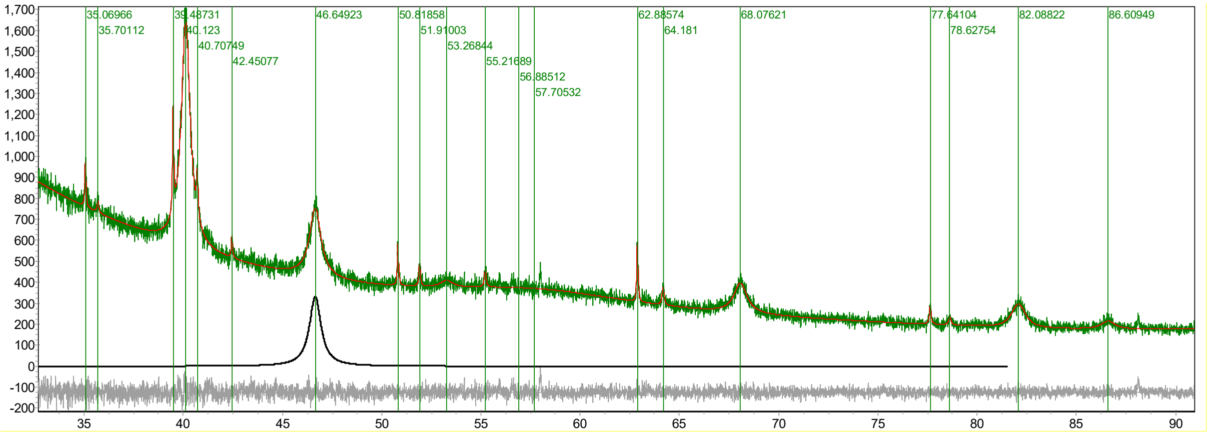

Figure S1. Experimental diffraction pattern of Pd@**5b** sample (green curve) and theoretical calculated curve (red curve). Solid vertical lines show the positions of the interference peaks corresponding to all crystalline phases, including the crystalline form of palladium Palladium, syn., Code no. 00-00-1201. The gray curve is the residual difference curve.

Table S1. The sizes of palladium crystallites, calculated from the parameters of diffraction peaks, for Pd@**5b** sample

**Miller indices**	**111**	**200**	**220**	**311**	**222**
**Angle 2θ, º**	40.123(2)	46.649(5)	68.076(9)	82.088(9)	86.61(3)
**CrySizeL (nm)**	17.8(2)	14.1(7)	13.8(9)	17.6(8)	14.7(8)
**LVol-IB (nm)**	11.4(4)	8.9(7)	8.8(9)	9.8(8)	9.4(9)
**Lvol-FWHM (nm)**	15.9(3)	12.6(7)	12.3(9)	12.7(9)	13.1(9)

R_{wp}	5.37%
R_{exp}	5.08%

Small angle X-Ray scattering (SAXS). Scattering patterns were obtained for the samples at 23°C in an evacuated chamber. The measurements were performed in transition mode with the use of glass capillaries filled by liquid sample and ethanol. The capillaries (2 mm diameter) were sealed and put into evacuated chamber by means of the holders. For each sample 10 experiments were performed with 5000 sec data collection and 1000 sec for absorption correction, allowing to control stability of the samples and quality of the experiments. The results of the experiments are averaged, so that the total time of each experiment was equal to 5000 sec. The data were corrected for background scattering and absorption of the samples. The 2D scattering patterns were integrated using the SAXS program package.[4] Calculation of structural parameters, simulation, and graphical representation of the results were performed using SASView[5] and PRIMUS[6] program packages.
The two-dimensional small-angle scattering patterns obtained for the two samples correspond to the scattering of isotropic heterogeneous systems, which follows from the uniform intensity distribution around the primary X-ray beam in Figure S2a. Integration of two-dimensional diffraction patterns made it possible to obtain curves of the dependence of small-angle scattering of the samples. For comparison, Figure S2b shows the diffraction patterns of scattering by ethanol and two samples without background subtraction.

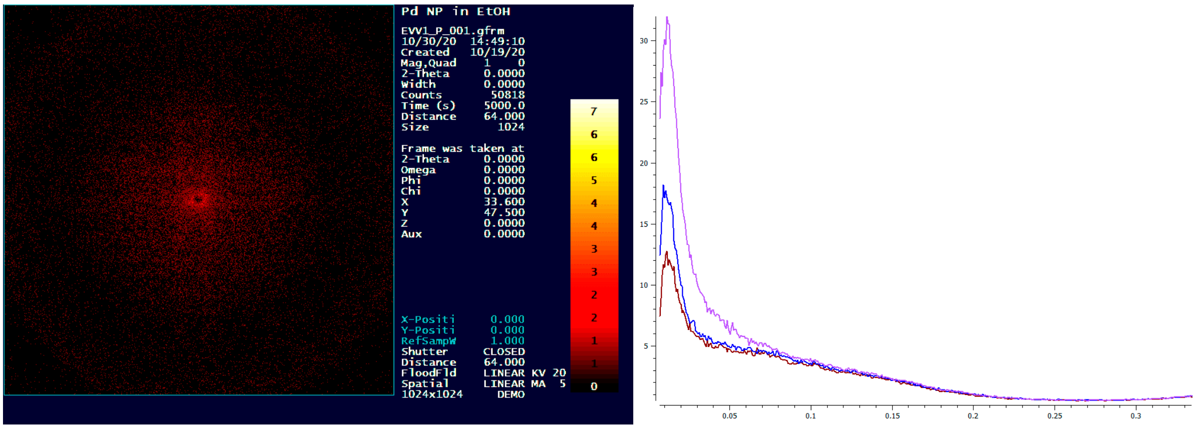

a b

Figure S2. (a) Screenshots of the 2D small-angle scattering pattern of the Pd@**10b** sample; (b) SAXS diffraction intensity profiles (Intensity vs. s) at 23 °C: sample Pd@**10b** (violet curve), sample Pd@**5b** (blue curve) and EtOH (brown curve), scattering vector $s = 4\pi \mathrm{Sin}\theta/\lambda$, λ is the wavelength of the incident X-ray beam

According to the small-angle X-ray scattering data, both samples are characterized as heterogeneous, with the presence of the randomly oriented particles in solutions, the dimensional characteristics of which correspond to the information area of the SAXS method (1 - 100 nm)[7]. The modelling of small-angle scattering was performed by calculating the scattering for a generalized Guinier/power law object.[8] This is an empirical model that can be used to determine the size and dimensionality of scattering objects, including asymmetric objects such as rods or platelets, and shapes intermediate between spheres and rods or between rods and platelets, and overcomes some of the deficiencies of the (Beaucage) Unified_Power_Rg model.[9] Figures S3 show the corresponding curves and fitting for two samples. Table S2 collects the calculated from the SAXS data parameters characterizing nanoparticles, such as radius of gyration (R_g), dimensionality parameters of the particles (S) and average diameter of the particles in a sphere-shaped model framework ($R_{sphere}=\sqrt{(5/3)\cdot R_g^2}$). The correctness of the fitting was ensured by minimizing the convergence parameters Chi^2.
The obtained values of the parameter S testify in favor of the spherical shape of the particles. The increased, in comparison with the data of powder diffraction, calculated particle sizes R_{sphere} may indicate

a more complex morphology of the resulting nanoparticles, in particular, the formation of double layers on their surface.

Table S2. Calculated parameters for the samples Pd@**10b** and Pd@**5b**

**Parameters**	Pd@**10b**	Pd@**5b**
**R_g, Å**	83.5 ± 2.2	109.4 ± 8.2
**S**	1.1277e-09	5.8395e-09
**R_{sphere}, Å**	107.8	141.1
Chi^2	0.0828	0.0402

(R_g is the radius of gyration of the particles. R_{sphere} is the average diameter of the particles in a sphere-shaped model framework ($R_{sphere} = \sqrt{(5/3)\cdot R_g^2}$).

S - dimensionality parameter: for globular objects (such as spheres) S=0, for 2D symmetry (such as for rods) S=1, and for 1D symmetry (such as for lamellae or platelets) S=2.

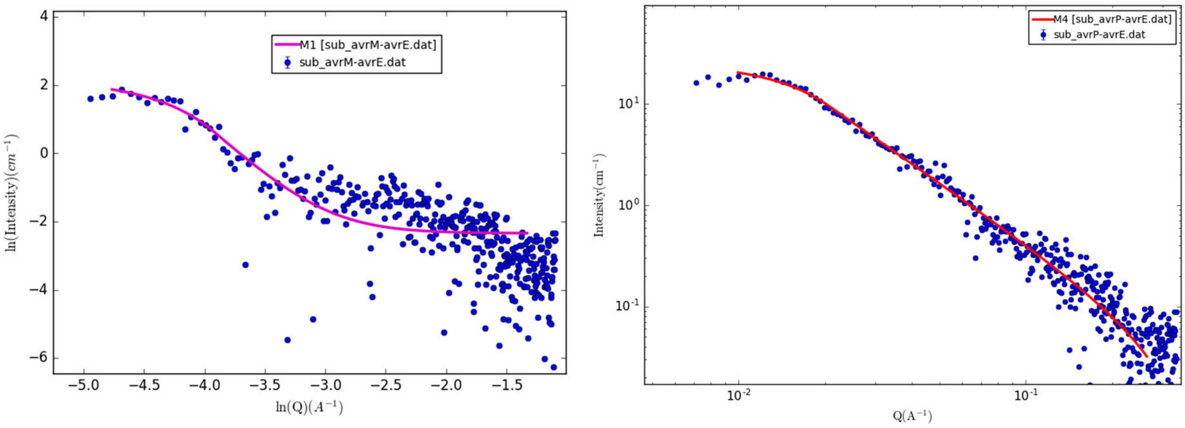

Figure S3. The fitting of experimental SAXS curve for samples Pd@**5b** (a) and Pd@**10b** (b) in the case of Guinier-Porod model *(*in duble logarithmic scale*, Ln(I) vs Ln(s), e*xperimental points – circles, solid line – calculated curves*).*

General procedure for Suzuki cross-coupling

To the fresh solution of colloidal Pd 0.157 g (0.50 mmol) of 1,3,5-tribrombenzene, 0.276 g (2.25 mmol) of phenyl boronic acid, 0.128 g (2.25 mmol) of potassium hydroxide were added. Reaction mixture was stirred over 7 h at 30 °C. Organic compounds were extracted with 9 ml toluene and analyzed by GC-MS.

[1] DIFFRAC Plus Evaluation package EVA, Version 11 (2005). User's Manual, Bruker AXS, Karlsruhe. Germany. - 258 p.

[2] TOPAS V3: General profile and structure analysis software for powder diffraction data. Technical Reference. Bruker AXS: Karlsruhe. Germany, 2005, 258 pp.

[3] TOPAS V3: General profile and structure analysis software for powder diffraction data. (2005). Technical Reference. Bruker AXS. Karlsruhe. Germany. 117 p.

[4] Small Angle X-ray Scattering. Version 4.0. Software Reference Manual, 2000, M86-E00005-0600, Bruker AXS Inc.

[5] SASView 3.0.0, University of Tennessee, 2009—2013 http://www.sasview.org

[6] P.V. Konarev, V. V. Volkov, A.V. Sokolova, M.H.J. Koch, D.I. Svergun, *J. Appl. Cryst*., 2003, **36**, 1277.

[7] L.A. Feigin, D.I. Svergun, Structure Analysis by Small-Angle X-Ray and Neutron Scattering, Plenum Press, New York, 1987.

[8] B. Hammouda, A new Guinier-Porod model, *J. Appl. Cryst*., 2010, **43**, 716-719.

[9] SASView 3.0.0, University of Tennessee, 2009—2013 http://www.sasview.org
